# Synthesis and Characterization of Benzene- and Triazine-Based Azo-Bridged Porous Organic Polymers

**DOI:** 10.3390/polym15010229

**Published:** 2023-01-01

**Authors:** Barbara Panić, Tea Frey, Mladen Borovina, Kristijan Konopka, Miro Sambolec, Ivan Kodrin, Ivana Biljan

**Affiliations:** Department of Chemistry, Faculty of Science, University of Zagreb, Horvatovac 102A, HR-10000 Zagreb, Croatia

**Keywords:** azo linkages, CO_2_ adsorption, DFT calculations, nitrogen-rich, porous organic polymers, triazine

## Abstract

Porous organic polymers incorporating nitrogen-rich functionalities have recently emerged as promising materials for efficient and highly selective CO_2_ capture and separation. Herein, we report synthesis and characterization of new two-dimensional (2D) benzene- and triazine-based azo-bridged porous organic polymers. Different synthetic approaches towards the porous azo-bridged polymers were tested, including reductive homocoupling of aromatic nitro monomers, oxidative homocoupling of aromatic amino monomers and heterocoupling of aromatic nitro monomers and a series of aromatic diamines of different lengths and rigidity. IR spectroscopy, ^13^C CP/MAS NMR spectroscopy, powder X-ray diffraction, elemental analysis, thermogravimetric analysis, nitrogen adsorption–desorption experiments and computational study were used to characterize structures and properties of the resulting polymers. The synthesized azo-bridged polymers are all amorphous solids of good thermal stability, exhibiting various surface areas (up to 351 m^2^ g^−1^). The obtained results indicated that the synthetic methods and building units have a pronounced effect on the porosity of the final materials. Reductive and oxidative homocoupling of aromatic nitro and amino building units, respectively, lead to 2D azo-bridged polymers of substantially higher porosity when compared to those produced by heterocoupling reactions. Periodic DFT calculations and Grand-canonical Monte Carlo (GCMC) simulations suggested that, within the used approximations, linear linkers of different lengths do not significantly affect CO_2_ adsorption properties of model azo-bridged polymers.

## 1. Introduction

Porous organic polymers (POPs) are crystalline or amorphous materials that can be constructed by connecting organic molecular building units through covalent bonds [1,2,3,4]. POPs are divided into several classes, including covalent organic frameworks (COFs), conjugated microporous polymers (CMPs), hyper-cross-linked polymers (HCPs), crystalline triazine frameworks (CTFs), porous polymer networks (PPNs), polymers of intrinsic microporosity (PIMs), porous aromatic frameworks (PAFs), etc., which differ in structural features and synthesis approaches. POPs have drawn great interest in recent years due to their high specific surface area, permanent and tunable porosity, high thermal and chemical stability and low density, which make them suitable for a range of applications, e.g., in gas storage and separation, catalysis, optoelectronics, sensing and drug delivery [5,6,7,8,9,10,11,12,13]. One of the most important potential applications of POPs is the capture of CO_2,_ which is the primary greenhouse gas whose elevated emissions into the atmosphere are closely related to global warming, sea level rising and ocean acidification [14,15]. CO_2_ capture capacity and selectivity could be improved by incorporating nitrogen-rich functionalities into the building units (e.g., imine, carbazole, triazine, benzimidazole, azo, etc.) that act as CO_2_-phylic sites [3,16,17,18,19,20,21,22]. Namely, CO_2_ possesses a higher quadrupole moment compared to most other gases, including N_2_, rendering the moieties that contain polar functional groups or heteroatoms CO_2_-phylic. This is particularly important given that a large Brunauer–Emmett–Teller (BET) surface area is not necessarily the dominant factor for high CO_2_ uptake capacity and that presence of heteroatoms can provide favorable interactions with CO_2,_ leading to enhanced CO_2_ adsorption and CO_2_/N_2_ selectivity [3,23,24]. A widely used method of providing a nitrogen-rich environment that enhances affinity for CO_2_ adsorption is the introduction of triazine units within the POPs [17,18,19,20,21,25]. Triazine-based POPs, besides possessing high nitrogen content, also exhibit high chemical and thermal stability, making them one of the most promising materials for practical applications in this field. Furthermore, recent studies indicated that azo groups are particularly well-suited for design of POPs, which could be used for highly selective gas separations [24,26,27,28,29,30,31]. It was reported that azo-bridged POPs show excellent CO_2_/N_2_ selectivity, which increases with rising temperature [24,26]. In fact, azo groups reject N_2_, making the framework N_2_-phobic while exhibiting high affinity towards polarizable CO_2_ molecules through dipole–quadrupole interactions. Azo-bridged POPs can be synthesized by using different synthetic routes and various building units. Commonly employed synthetic methods producing 2D or three-dimensional (3D) porous azo-bridged polymers include Zn- or NaBH_4_-mediated reductive homocoupling of aromatic nitro monomers [32,33], copper(I)-catalyzed oxidative homocoupling of aromatic amino monomers [27,28] and heterocoupling of aromatic nitro and amino monomers under basic conditions [24,26]. In general, the use of different synthetic pathways and building units can result in azo-bridged POPs with different structural and functional properties, such as surface area, pore size and volume and CO_2_ uptake capacity and selectivity. 

**Figure 1:**
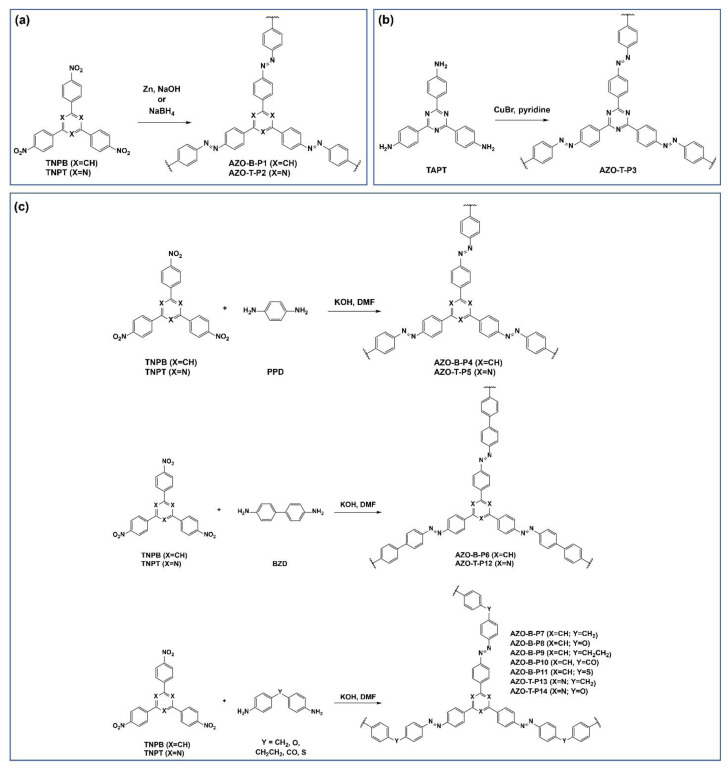
Synthesis of a series of benzene-(AZO-B-P) and triazine-based (AZO-T-P) azo-bridged polymers by (**a**) Zn- or NaBH_4_-mediated reductive homocoupling of aromatic nitro monomers (TNPB and TNPT); (**b**) copper(I)-catalyzed oxidative homocoupling of aromatic amino monomer (TAPT); (**c**) heterocoupling of aromatic nitro monomers (TNPB and TNPT) and various aromatic diamines under basic conditions.

In the present study, we synthesized a series of new 2D benzene- and triazine-based azo-bridged POPs by using different synthetic routes and a variety of aromatic nitro and amino building units. Specifically, we used 1,3,5-tris(4-nitrophenyl)benzene (TNPB) and 2,4,6-tris(4-nitrophenyl)-1,3,5-triazine (TNPT) as the aromatic nitro monomers, and 4,4′,4′’-(1,3,5-triazine-2,4,6-triyl)trianiline (TAPT) and several aromatic diamines differing in length and rigidity as aromatic amino monomers for synthesis of a total of fourteen benzene- (AZO-B-P) and triazine-based (AZO-T-P) azo-bridged polymers (Figure 1). Thorough characterization of structures and properties of the resulting POPs was performed by IR spectroscopy, ^13^C CP/MAS NMR spectroscopy, powder X-ray diffraction (PXRD), elemental analysis, thermogravimetric analysis (TGA) and nitrogen (N_2_) adsorption–desorption experiments. In addition, DFT calculations and GCMC simulations were conducted on selected model azo-bridged polymers.

## 2. Materials and Methods

### 2.1. General Information

All chemicals were used as received from suppliers. The course of the reactions was monitored by thin-layer chromatography (TLC) (Merck silica gel 60-F254-coated plates). The synthesized compounds were identified by solution ^1^H and ^13^C NMR spectroscopy, solid-state ^13^C CP/MAS NMR spectroscopy, IR spectroscopy, PXRD and elemental analysis. Solution-state ^1^H and ^13^C NMR spectra were recorded on a Bruker Ascend 400 MHz NMR spectrometer in DMSO-d_6_. Solid-state ^13^C CP/MAS NMR spectra were recorded on a Bruker Avance Neo 400 MHz NMR spectrometer or Bruker Avance Neo 600 MHz NMR spectrometer at spinning rates of 12 kHz and 15 kHz, respectively. IR spectra were recorded on a PerkinElmer UATR Two spectrometer in the spectral range between 4000 cm^–1^ and 400 cm^–1^ at a resolution of 4 cm^–1^, averaging 10 scans per spectrum. PXRD diffractograms were recorded on a Malvern Panalytical Aeris powder diffractometer in Bragg–Brentano geometry with PIXcel^1D^ detector. Thermogravimetric analysis was performed using a simultaneous TGA-DTA analyzer Mettler-Toledo TGA/DSC 3+. Samples were placed in alumina pans (70 μL), heated in flowing nitrogen (50 mL min^−1^) from 25 °C up to 800 °C at a rate of 10 °C min^−1^ and held in isothermal conditions for 15 min at 800 °C. Data collection and analysis were performed using the program package STARe Software 16.40 MettlerToledo GmbH (Greifensee, Switzerland). The specific surface area was determined from N_2_ gas adsorption–desorption data obtained with Micromeritics ASAP-2000 at 77 K. Prior to analysis, samples were degassed at 150 °C under a dynamic vacuum of 7 mPa. The adsorption data were used to calculate the surface area with the BET model, while the pore size distribution was determined with the Barrett–Joyner–Halenda (BJH) method.

### 2.2. General Synthetic Procedures

Azo-bridged polymers were synthesized by three different methods: (a) reductive homocoupling of aromatic nitro compounds (TNPB and TNPT) using Zn or NaBH_4_ as reducing agent (AZO-B-P1 and AZO-T-P2); (b) oxidative homocoupling of aromatic amino compound (TAPT) with CuBr as an oxidizing agent (AZO-T-P3); (c) condensation reactions of various aromatic nitro compounds and aromatic diamines under basic conditions (AZO-B-P4–AZO-T-P14) (Figure 1). All diamines, 1,4-phenylenediamine (PPD), benzidine (BZD), 4,4′-diaminodiphenylmethane, 4,4′-oxydianiline, 4,4′-ethylenedianiline, 4,4′-diaminobenzophenone and 4,4′-diaminodiphenyl sulfide, were purchased from the suppliers. 1,3,5-Tris(4-nitrophenyl)benzene (TNPB) [34], 2,4,6-tris(4-nitrophenyl)-1,3,5-triazine (TNPT) [35] and 2,4,6-tris(4-aminophenyl)-1,3,5-triazine (TAPT) [35] were synthesized by the procedures described in the literature.

#### 2.2.1. Synthesis of AZO-B-P1

AZO-B-P1 was prepared by a similar procedure described in the literature [32]. TNPB (500 mg, 1.13 mmol) was dissolved in a mixture of 7 mL tetrahydrofuran (THF) and 8 mL *N*,*N*-dimethylformamide (DMF). NaOH solution (723 mg NaOH in 1.7 mL of de-ionized water) and zinc powder (665 mg) were added to the reaction mixture. The reaction mixture was heated at 65 °C for 36 h. After cooling to room temperature, the mixture was poured into 100 mL of 2M HCl and stirred for 1 h. The solid was filtered off and washed with water, acetone and THF. After drying at 140 °C under vacuum for 5 h, 394 mg of a red solid product was obtained (yield 50%). Elemental analysis: 69.93%C (calc. 83.46), 10.14%N (calc. 12.17).

#### 2.2.2. Synthesis of AZO-T-P2

AZO-T-P2 was synthesized by a similar procedure described in the literature [33]. In the mixture of TNPT (0.5 g, 1.13 mmol) and DMF (30 mL), a solution of NaBH_4_ (0.128 g, 3.39 mmol) and DMF (20 mL) was added dropwise. The reaction mixture was heated at 85 °C for 24 h and then filtered and washed with DMF, HCl, water and THF. After drying at 140 °C under vacuum for 5 h, 295 mg of an orange solid product was obtained (yield 37%). Elemental analysis: 70.55%C (calc. 72.40), 22.95%N (calc. 24.12).

#### 2.2.3. Synthesis of AZO-T-P3

AZO-T-P3 was synthesized by a similar procedure described in the literature [27]. TAPT (100 mg, 0.28 mmol) was dissolved in 22 mL solvent mixture of THF/toluene = 1:1. In the reaction mixture were added CuBr (20.2 mg, 0.141 mmol) and pyridine (80.4 mg, 1.02 mmol). The mixture was stirred 24 h at room temperature, 12 h at 60 °C and 12 h at 80 °C and then filtered and washed with THF and water. The solid product was soaked in 100 mL of 4M HCl for 24 h and then filtered and washed with water, NaOH (200 mL, 1 M), water and ethanol. After drying at 140 °C under vacuum for 5 h, 103 mg of a dark brown solid was obtained (yield 52%). Elemental analysis: 60.72%C (calc. 72.40), 18.84%N (calc. 24.12).

#### 2.2.4. Synthesis of AZO-B-P4

AZO-B-P4 was synthesized by a similar procedure described in the literature [24]. TNPB (500 mg, 1.13 mmol), PPD (184 mg, 1.70 mmol), DMF (50 mL) and KOH (634 mg, 11.3 mmol) were added in double-necked flask and heated to reflux under N_2_ atmosphere. After 24 h, the reaction mixture was cooled to room temperature, poured in 300 mL of distilled water and stirred for 1 h. The reaction mixture was filtered off and washed with hot distilled water, acetone and THF. After drying at 140 °C under vacuum for 5 h, 396 mg of a black solid was obtained (yield 78%). Elemental analysis: 57.84%C (calc. 80.16), 9.59%N (calc. 15.58).

The other AZO-B-Ps and AZO-T-Ps were prepared by similar procedures (Supplementary Materials).

### 2.3. Computational Methods

The initial geometries of AZO-B and AZO-T were taken from our previous study [36] and changed in GaussView (PBC tool) and VESTA to construct other systems (AZO-B-PPD, AZO-B-BZD, AZO-T-PPD and AZO-T-BZD) with PPD and BZD linkers [37,38]. The space group symmetry was checked by PLATON [39]. PBE functional [40], Grimme’s D3 correction [41] and triple-zeta basis set pob-TZVP-rev2 [42] were used for periodic density functional theory (DFT) calculations in CRYSTAL17 [43]. The input files for CRYSTAL17 were created with cif2cell package [44]. Full optimization of both atom coordinates and unit cell parameters were performed with default convergence criteria. Total energy convergence was set to 10^−7^ and truncation criteria for the calculations of Coulombs and exchange integrals increased to (8 8 8 8 16) for SCF calculations. The reciprocal space was sampled using 2 × 2 × 8 Pack-Monkhorst k-point mesh. The lattice parameters of the DFT-optimized structures are given in Table S1 of the Supplementary Materials.

The net atomic charges were calculated using the REPEAT method [45] from the electron densities calculated by CRYSTAL17. The required cube files with electrostatic potential values were generated by CRYSTAL17. The GCMC simulations were performed with the RASPA code [46]. The site–site Lennard-Jones (LJ) potential and Coulombic interactions were used together with Lorentz–Berthelot mixing rules for the LJ interactions between different atoms to represent interactions between gas molecules and the framework. A three-site model was used to represent the gas molecules, CO_2_ and N_2_, within the TraPPE force field [47]. Other atoms from the framework were modeled using DREIDING force field [48]. Default cut-off values suggested by RASPA code were used for LJ and the short-range part of the Coulombic interactions, while the long-range was evaluated by Ewald summation method with a default relative precision of 10^−6^. The grand canonical Monte Carlo (GCMC) simulation was performed to obtain single-component adsorption isotherms of N_2_ and CO_2_ at 298 K. Pressure was converted to fugacity using the Peng–Robinson equation of state and further used to calculate the chemical potential. A total number of unit cells, 2 × 2 × 8, was used to describe a valid GCMC simulation cell. All the perpendicular cell lengths were larger than twice the default cut-off distance of 12 Å. Framework atoms were frozen and four different MC moves of gas molecules (translation, rotation, reinsertion and swap) were allowed during simulations. More than 10^6^ cycles were used for the equilibration and production phases.

## 3. Results and Discussion

### 3.1. Synthesis of Azo-Bridged Polymers

Benzene- and triazine-based azo-bridged polymers (AZO-B-Ps and AZO-T-Ps, respectively) were prepared using different synthetic approaches adopted from previously reported procedures for azo POPs [24,26,27,28,32,33]. For synthesis of AZO-B-P1 and AZO-T-P2, the corresponding starting aromatic nitro monomers, TNPB and TNPT, were subjected to Zn- and NaBH_4_-induced reductive homocoupling reactions [32,33], respectively (Figure 1a). Triazine-based polymer AZO-T-P3 was synthesized by copper(I)-catalyzed oxidative homocoupling of aromatic amino monomer, TAPT, by following the previously reported procedure for its benzene analogue [27] (Figure 1b). To investigate the effect of different linear linkers on the porosity properties of benzene- and triazine-based azo-bridged polymers, we also employed metal catalyst-free direct heterocoupling of aromatic nitro monomers, TNPB and TNPT, and various aromatic diamines [24,26], including p-phenylenediamine (AZO-B-P4 and AZO-T-P5), benzidine (AZO-B-P6 and AZO-T-P12)**,** 4,4′-diaminodiphenylmethane (AZO-B-P7 and AZO-T-P13)**,** 4,4′-oxydianiline (AZO-B-P8 and AZO-T-P14)**,** 4,4′-ethylenedianiline (AZO-B-P9), 4,4′-diaminobenzophenone (AZO-B-P10) and 4,4′-diaminodiphenyl sulfide (AZO-B-P11), under basic conditions (Figure 1c)**.** The molar ratio of TNPB and TNPT to the corresponding diamine was set to 3:2. All reactions afforded solid products insoluble in common organic solvents, such as acetone, THF, dichloromethane, DMF and DMSO, suggesting the formation of cross-linked polymer networks.

### 3.2. Characterization of Azo-Bridged Polymers

**Figure 2:**
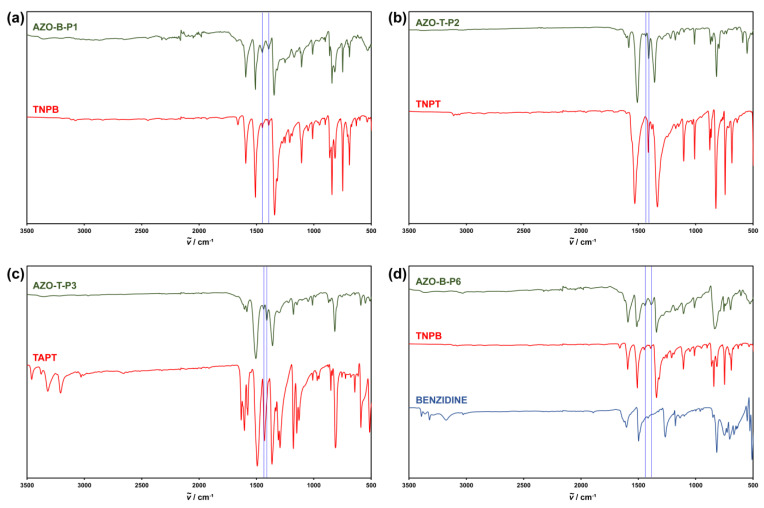
Comparison of representative FTIR spectra of (**a**) AZO-B-P1 polymer and starting nitro monomer (TNPB); (**b**) AZO-T-P2 polymer and starting nitro monomer (TNPT); (**c**) AZO-T-P3 polymer and starting amino monomer (TAPT); (**d**) AZO-B-P6 polymer and starting nitro and amino monomers (TNPB and benzidine, respectively). Signals attributed to the stretching vibrations of the azo (–N=N–) group in polymers are marked with vertical lines.

Formation of azo-bridged polymers was verified by IR spectroscopy, ^13^C CP/MAS NMR spectroscopy, PXRD and elemental analysis. Figure 2 shows a comparison of representative FTIR spectra of polymers AZO-B-P1, AZO-T-P2, AZO-T-P3 and AZO-B-P6, prepared by different synthetic routes, and respective starting nitro and/or amino monomers. The spectra of polymers revealed the presence of bands around 1450 cm^–1^ and 1400 cm^–1^, which can be attributed to the asymmetric stretching vibrations of the azo (–N=N–) group (Figure 2, marked with vertical lines). Similar peaks can also be observed in FTIR spectra of other synthesized azo-bridged polymers (Figure S1). In addition, bands at 1520 cm^–1^ and 1350 cm^−1^, assigned to asymmetric and symmetric N–O stretching bands of unreacted terminal nitro groups, could be observed in the FTIR spectra of polymers synthesized by Zn- and NaBH_4_-mediated reductive homocoupling of TNPB and TNPT, and by heterocoupling of TNPB and TNPT with various diamines (Figure 2a,b,d and Figure S1). In most polymers, we could detect the residual signals located in the 3400–3300 cm^–1^ region, which probably belong to N–H stretching vibrations and suggest the presence of terminal amino groups. The IR spectra of triazine-based azo polymers also showed bands in the 1600–1300 cm^–1^ region and at 800 cm^–1^, which could be attributed to the stretching vibrations and the breathing modes of the triazine units, respectively. 

**Figure 3:**
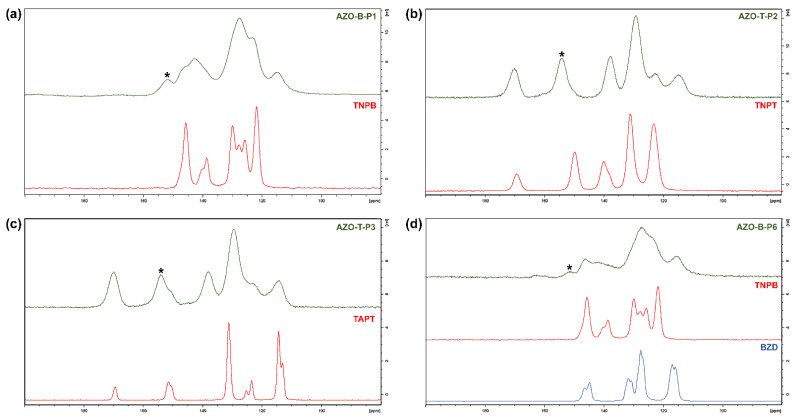
Comparison of representative ^13^C CP/MAS NMR spectra of (**a**) AZO-B-P1 polymer and starting nitro monomer (TNPB); (**b**) AZO-T-P2 polymer and starting nitro monomer (TNPT); (**c**) AZO-T-P3 polymer and starting amino monomer (TAPT); (**d**) AZO-B-P6 polymer and starting nitro and amino monomers (TNPB and benzidine, respectively). Marked with asterisk (*) is the signal of carbon directly bonded to the azo group (–C–N=N–).

Although FTIR spectra of polymers indicated formation of azo linkages, this needed further confirmation since it is difficult to identify azo group solely by IR spectroscopy. Therefore, we acquired and compared ^13^C CP/MAS NMR spectra of starting compounds and obtained polymeric products (Figure 3 and Figure S2). Comparison between the ^13^C CP/MAS NMR spectra of AZO-B-Ps and AZO-T-Ps and amino and nitro building units (Figure 3 and Figure S2) clearly supported successful formation of azo polymers from the corresponding monomers. The most prominent feature in the ^13^C CP/MAS NMR spectra of the polymers was the appearance of a new signal around δ = 150 ppm (marked with asterisk in Figure 3), belonging to the carbon directly bonded to the azo group (–C–N=N–), which confirmed the formation of azo bonds. The additional signals in the spectra of products could be assigned to the other carbon atoms in building units of azo polymers. Thus, in the spectra of AZO-B-Ps and AZO-T-Ps, we could observe aromatic carbon signals in different chemical environments mostly located in the range δ = 110–155 ppm. The spectra of AZO-T-Ps (Figure 2b,c and Figure S2) also displayed triazine carbon signal around δ = 170 ppm, confirming the presence of triazine units. 

**Figure 4:**
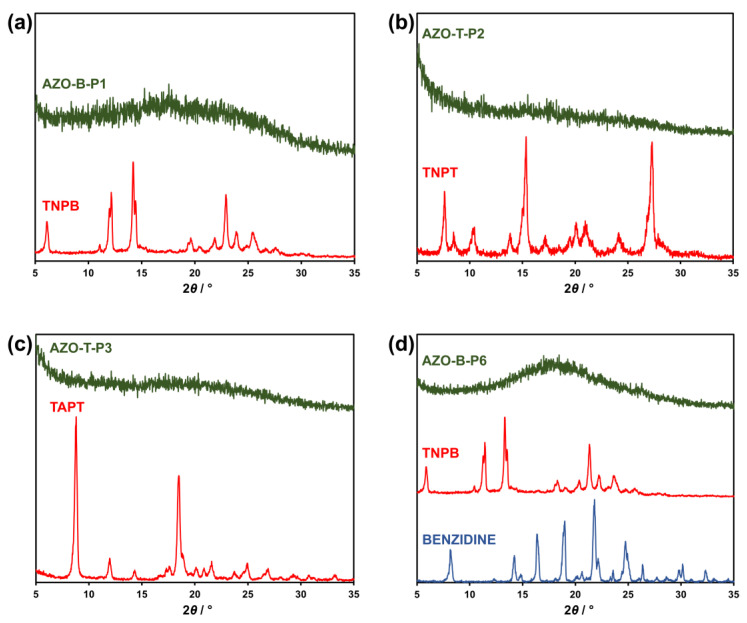
Comparison of representative PXRD patterns of (**a**) AZO-B-P1 polymer and starting nitro monomer (TNPB); (**b**) AZO-T-P2 polymer and starting nitro monomer (TNPT); (**c**) AZO-T-P3 polymer and starting amino monomer (TAPT); (**d**) AZO-B-P6 polymer and starting nitro and amino monomers (TNPB and benzidine, respectively).

Comparison of PXRD patterns of starting nitro and amino compounds and obtained polymers additionally corroborated the results of FTIR and ^13^C CP/MAS NMR spectroscopy, suggesting the formation of new products (Figure 4 and Figure S3). In contrast to starting compounds, which provided sharp diffraction peaks, indicating they are crystalline solids, PXRD of all fourteen polymeric products displayed broad diffraction peaks, pointing to their amorphous nature with no long-range order. This agrees with the literature data available for similar azo-bridged polymers, which are also amorphous because of the irreversible formation of the azo bond [24,26,27,28,29,30,31].

Elemental analysis data of AZO-B-Ps and AZO-T-Ps revealed some deviations from the theoretical values, which is commonly observed in POPs and is attributed to incomplete polymerization and adsorption of moisture [27,28,49].

Thermal stability was investigated using TGA by heating the AZO-B-Ps and AZO-T-Ps samples up to 800 °C at a heating rate of 10 °C min^–1^ in N_2_ atmosphere. TGA traces showed that benzene-based azo polymers, AZO-B-Ps, are stable up to approximately 220 °C, at which point they begin to gradually lose mass, and, at around 250 °C, they rapidly decompose (Figure S4). Azo polymers containing triazine central unit, AZO-T-Ps, mostly showed higher thermal stability in comparison to AZO-B-Ps and remained stable up to around 250 °C, when they begin to gradually lose mass (<5%) until around 350 °C (AZO-T-P14) to 450 °C (AZO-T-P2), at which point they start to decompose more rapidly. The highest thermal stability was observed for AZO-T-P2. 

### 3.3. Porosity Properties of Azo-Bridged Polymers

**Figure 5:**
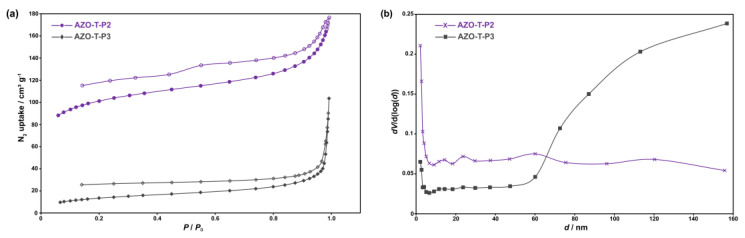
(**a**) N_2_ adsorption–desorption isotherms of AZO-T-P2 and AZO-T-P3 measured at 77 K. The adsorption and desorption isotherms are depicted with filled and open markers, respectively; (**b**) pore size distributions of AZO-T-P2 and AZO-T-P3.

Porosity properties of AZO-B-Ps and AZO-T-Ps were investigated by measuring the N_2_ adsorption–desorption isotherms at 77 K (Figure 5a). Prior to the analysis, all samples were degassed at 150 °C under vacuum. The specific surface areas were estimated by using the BET model and ranged from 0.05 to 351 m^2^ g^−1^ (Table 1). The highest surface area of 351 m^2^ g^−1^ was obtained for AZO-T-P2 synthesized by NaBH_4_-mediated reductive homocoupling of aromatic nitro monomer, TNPT. Notably, this value is significantly higher in comparison to the specific surface area of AZO-T-P3 (50.8 m^2^ g^−1^), which is composed of the same building units but synthesized by copper(I)-catalyzed oxidative homocoupling of aromatic amino monomer, TAPT. According to the obtained pore size distribution (Figure 5b), AZO-T-P3 could be characterized as a mesoporous material with a proportion of pores larger than 50 nm. There is a certain amount of micropores, but not large enough to show a significant volume of micropores. On the other hand, AZO-T-P2 showed a greater extent of micropores (Figure 5b) but could also be characterized as a mesoporous material. The recorded adsorption–desorption isotherms supported this observation. Both AZO-T-P2 and AZO-T-P3 showed type IV isotherms according to the IUPAC classification [50], which is characteristic of mesoporous materials. The observed adsorption hysteresis is expected in type IV isotherms and is related to capillary condensation and evaporation in the mesopores. As known from the literature, the shape of the adsorption hysteresis loop correlates with the pore size distribution, pore geometry and its connectivity [51]. The H4 hysteresis loop present in AZO-T-P2 is commonly associated with narrow slit-shaped pores, while the H3 hysteresis loop visible in AZO-T-P3 is observed in materials with slit-shaped pores. An increase in nitrogen uptake at higher relative pressures (above 0.9) indicated the presence of interparticular voids in the networks of AZO-T-P2 and AZO-T-P3 [24,29,52]. 

Differences in the obtained surface area values of AZO-T-P2 and AZO-T-P3 emphasized the important role of the synthetic route on the porosity of the final material. This is also evident from the comparison of BET surface areas of AZO-B-P1 (2.40 m^2^ g^−1^), prepared by Zn-induced reductive homocoupling of aromatic nitro monomer, TNPT, and ALP-4 (223 m^2^ g^−1^ to 862 m^2^ g^−1^) [27], synthesized by oxidative homocoupling of aromatic amino monomer, TAPT, in the presence of copper(I) bromide. In an attempt to prepare AZO-B-P1 with a higher surface area, we also tested NaBH_4_-mediated reductive homocoupling of TNPB monomer. However, the reaction was unsuccessful, and we could only detect starting TNPB in the final reaction mixture. Azo-bridged polymers with benzene and triazine central units (AZO-B-P4–AZO-T-P14) prepared by heterocoupling of aromatic nitro monomers, TNPB and TNPT, and different aromatic diamines exhibited very low BET surface area values (Table 1). Noteworthy, this is in sharp contrast to 3D azo-bridged polymers synthesized by coupling of tetrahedral aromatic nitro monomers and aromatic diamines, which mostly showed moderate to high porosities [24,26]. It has been reported earlier that POPs constructed from 3D building units in general possess higher surface areas in comparison to those produced by using only 2D building units [22,32]. Lower surface area values observed for AZO-B-P4–AZO-T-P14 containing linear linkers in comparison to, e.g., AZO-T-P2 and AZO-T-P3, in which triphenyltriazine units are directly connected, could be due to an increased degree of conformational freedom in the former case, allowing for greater interpenetration and more efficient space filling in the networks [53,54,55].

### 3.4. Computational Studies of Azo-Bridged Polymers

In our recently published paper on the computational study of structural and adsorption properties of benzene- and triazine-based polymers with azo, azoxy and azodioxy linkages, we proposed a procedure based on three complementary methods to acquire guidelines for the future synthesis of promising porous organic materials [36]. Aside from the calculation of binding energies and comparison of the best interaction sites based on the calculated electrostatic potential values, we also performed periodic DFT calculations and GCMC simulations. These results provide us with additional information about the structural properties resulting in better adsorption of CO_2_ in nitrogen–nitrogen linked POPs. First, we assumed packing of azo-, azoxy- and azodioxy-linked polymers as in similar systems (such as those with imine bonds) for which crystal structures were determined. Second, we studied two arrangements of 2D layers with hexagonal pores, AA and AB stacking. AA-stacked layers were energetically more favorable but showed lower CO_2_ uptake compared to AB-arranged layers.

**Figure 6:**
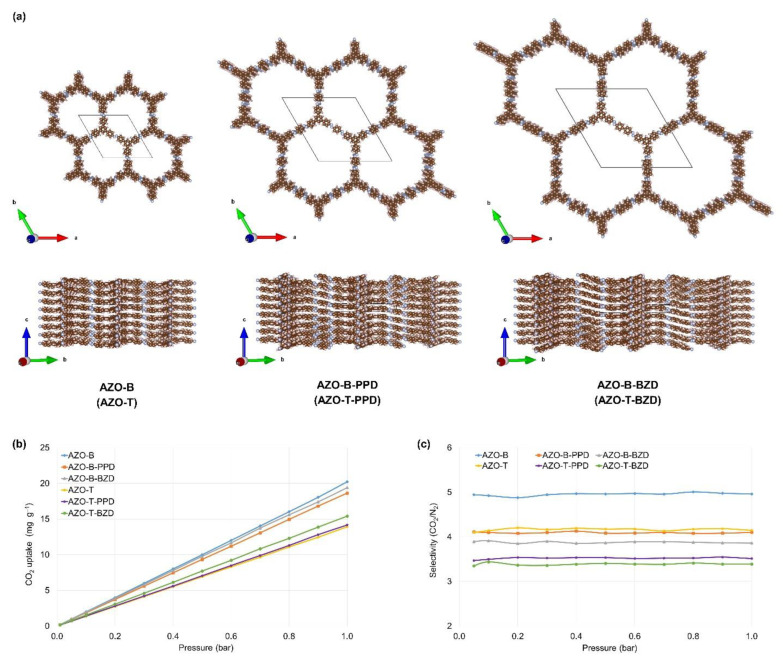
(**a**) Optimized geometries (PBE-D3/pob2-TZVP-rev2) of AZO-B, AZO-B-PPD and AZO-B-BZD with eclipsed (AA-stacked) geometries of 2D layers shown along the *c* and *a* unit cell vectors. Triazine-based compounds (labels in parentheses) have analogous structures (more details in Figure S5). Unit cells are represented by grey lines. (**b**) CO_2_ adsorption isotherms simulated at 298 K. (**c**) CO_2_/N_2_ selectivities calculated for a molar ratio of 15:85 at 298 K.

Compounds **1** and **4** from that study correspond to AZO-B-P1 and AZO-T-P2/P3, respectively, shown in Figure 1a, b. Azo-bridged POPs investigated experimentally in this study were amorphous solids; thus, it was difficult to predict their structures. Here, we focused only on the geometries of six azo-bridged benzene- and triazine-based POPs. In AZO-B and AZO-T, trigonal building units were directly connected by azo bonds, while linear linkers (PPD and BZD) bridged the connectors in AZO-B-PPD, AZO-T-PPD, AZO-B-BZD and AZO-T-BZD. Other systems with bent linkers were not investigated because of greater conformational flexibility and possible interweaving of chains. The energetically most favorable AA configurations with eclipsed 2D layers (Figure 6a and Figure S5, Table S1) were predicted, simply to avoid all the other unnecessary combinations, while changing only one parameter at a time, the length of the linear linker.

For perfectly arranged AA-stacked layers, pore size diameters (between 18 and 36 Å, Figure S6) and specific surface areas (from 1828 to 2306 m^2^ g^−1^, Table S2) increase when going from directly connected building units in AZO-B and AZO-T to those bridged by PPD and BZD linkers. The calculated pore size diameters indicated the uniform distribution of micropores, while experiments also showed the presence of mesopores. The value of specific surface area calculated for AZO-T (1828 m^2^ g^−1^) differs from the experimentally determined BET surface areas for amorphous AZO-T-P2 (351 m^2^ g^−1^) and AZO-T-P3 (50.8 m^2^ g^−1^). However, the calculated values are comparable to those determined for the same building (triphenyltriazine) units connected by topologically very similar imine bonds in AA-arranged crystalline TPT-COF-6 (1535 m^2^ g^−1^) [56]. That means the computational procedures work decently, but it is difficult to expect this type of 2D layer arrangement for amorphous azo polymers investigated in this paper.

The effect of different synthetic procedures on the sizes of pores and CO_2_ uptake is more than obvious in AZO-B. The experimentally determined BET surface area of 2.4 m^2^ g^−1^ in AZO-B-P1 differs from the previously found values for a similar system ALP-4 (from 223 to 862 m^2^ g^−1^), where they depend on stepwise increase in temperature during synthesis [28]. These values are still lower than those calculated for AZO-B (1957 m^2^ g^−1^). The reported CO_2_ uptake of 81 mg g^−1^ at 1 bar and 298 K and CO_2_/N_2_ selectivity of 26 at molar ratio 15:85 [28] differ from the values calculated for eclipsed 2D layers in AZO-B (20 m^2^ g^−1^, Figure 6b,c). However, introduction of more polar linkages, such as azodioxy, in place of azo bonds increases CO_2_ uptake from 20 to 30 m^2^ g^−1^ in AZO-B, respectively [36]. Our previous study also showed that staggered configurations (staggered AB), although energetically less favorable, can promote higher CO_2_ uptakes and selectivities [36]. Different arrangements of 2D layers (e.g., eclipsed, staggered, serrated or inclined) can affect the targeted properties of material and should be considered in future studies [57,58].

Although higher values of CO_2_ uptake (for about 5 m^2^ g^−1^) are found in AZO-B compared to AZO-T, introduction of linear linkers (such as PPD and BZD) does not have a great impact on adsorption. However, CO_2_/N_2_ selectivity is slightly increased in compounds with directly attached building units (AZO-B and AZO-T). This computational analysis was focused only on linear linkers to keep it simple. The effect of other less conformationally rigid linkers should be tested in the future, especially in systems showing a greater tendency to be crystalline and thus more easily compared to experimental data.

## 4. Conclusions

We have synthesized fourteen new 2D azo-bridged polymers based on benzene and triazine building units by using different synthetic approaches and investigated their structural, thermal and porosity features. The prepared polymers are all amorphous solids of good thermal stability, with the highest thermal stability observed for triazine-based polymer AZO-T-P2. The obtained results indicated that the synthetic methods and building units have a significant effect on the porosity of the final materials. Specifically, the highest BET surface areas of 351 and 50.8 m^2^ g^−1^ were observed for the azo-bridged polymers with triazine central units, AZO-T-P2 and AZO-T-P3, prepared by NaBH_4_-mediated reductive homocoupling of nitro monomer and copper(I)-catalyzed oxidative homocoupling of amino monomer, respectively. Introduction of linear linkers utilizing heterocoupling reactions of aromatic nitro monomers containing benzene or triazine central units and different aromatic diamines resulted in azo polymers exhibiting very low BET surface areas, which is in contrast to previously reported azo porous systems prepared using 3D nitro building units. Although all the azo-bridged polymers synthesized here were characterized as amorphous solids, periodic DFT calculations and GCMC simulations on model systems can guide us in design of new functional materials for selective adsorption of CO_2_ by examination of the effect of different nitrogen–nitrogen linkages (e.g., azo, azoxy and azodioxy), various linkers and 2D layer stacking modes on the materials’ structural and adsorption properties. According to this computational study, introduction of linear linkers of different lengths does not significantly affect adsorption properties within the used approximations.

Overall, in this work, we identified new triazine-based azo-bridged POPs, AZO-T-P2 and AZO-T-P3, which could be promising candidates for selective CO_2_ capture. Further experimental investigations, including their CO_2_ adsorption capacities and CO_2_/N_2_ selectivities, are underway in our laboratory. 

## Figures and Tables

**Table 1:** BET surface areas (S_BET_) of AZO-B-Ps and AZO-T-Ps.

Sample	*S*_BET_ (m^2^ g^−1^)
AZO-B-P1	2.40
AZO-T-P2	351
AZO-T-P3	50.8
AZO-B-P4	0.74
AZO-T-P5	0.44
AZO-B-P6	0.43
AZO-B-P7	0.42
AZO-B-P8	0.83
AZO-B-P9	0.60
AZO-B-P10	0.63
AZO-B-P11	0.41
AZO-T-P12	0.05
AZO-T-P13	0.20
AZO-T-P14	0.45

## Data Availability

The synthesis, spectroscopic, thermal, PXRD, N_2_ adsorption–desorption and computational data are provided as figures and tables and are included in this paper.

## Supplementary Materials

The following supporting information can be downloaded at: https://www.mdpi.com/article/10.3390/polym15010229/s1, General synthetic procedures; Figure S1: FT-IR spectra of (a) starting aromatic nitro and amino monomers and (b) azo-bridged polymers; Figure S2: Comparison of ^13^C CP/MAS NMR spectra of azo-bridged polymers and corresponding starting aromatic nitro and amino monomers; Figure S3: PXRD diffractograms of (a) starting aromatic nitro and amino monomers and (b) azo-bridged polymers; Figure S4: TGA (black) and DSC (red) curves of azo-bridged polymers; Table S1: Unit cell parameters (*a*, *b*, *c*, α, β and γ) of the optimized geometries (PBE-D3/pob-TZVP-rev2) of benzene-based (AZO-B) and triazine-based (AZO-T) model compounds; Table S2: Calculated framework properties of the AA stacked 2D layered compounds (framework density, available pore volume and average surface area) and the size of the simulation box used in the GCMC calculations; Figure S5: Optimized geometries (PBE-D3/pob2-TZVP-rev2) of AZO-T, AZO-T-PPD and AZO-T-BZD with eclipsed (AA-stacked) geometries of 2D layers shown along the c and a unit cell vectors). Unit cells are represented by grey lines; Figure S6: Pores size distribution of AA stacked configurations; Figure S7: N_2_ adsorption isotherms simulated at 298 K.
