# Supplementary material for: Synthesis and Characterization of Benzene- and Triazine-Based Azo-Bridged Porous Organic Polymers
Source: Polymers (Basel). 2023 Jan 1;15(1):229. doi: 10.3390/polym15010229 (PMC9824540; doi:10.3390/polym15010229)
Supplement: Supplementary file 1 [file polymers-15-00229-s001.zip › polymers-2132846-supplementary.pdf]

Supplementary Materials

Contents

Contents 1

1. General synthetic procedures 2

    1.1. Synthesis of AZO-B-P1−AZO-T-P14 3

2. FT-IR spectra 8

3. ^{13}C CP/MAS NMR spectra 9

4. Powder X-ray diffraction 13

5. Thermogravimetric analysis 14

6. Computational studies of azo-bridged polymers 20

1. General synthetic procedures

Azo-bridged polymers were synthesized by three different methods: (a) reductive homocoupling of aromatic nitro compounds (TNPB and TNPT) using Zn or NaBH4 as reducing agent (AZO-B-P1 and AZO-T-P2); (b) oxidative homocoupling of aromatic amino compound (TAPT) with CuBr as an oxidizing agent (AZO-T-P3), (c) condensation reactions of various aromatic nitro compounds and various aromatic diamines under basic conditions (AZO-B-P4 – AZO-T-P14). All diamines, 1,4-phenylenediamine (PPD), benzidine (BZD), 4,4'-diaminodiphenylmethane, 4,4'-oxydianiline, 4,4'-ethylenedianiline, 4,4'-diaminobenzophenone and 4,4'-diaminodiphenyl sulfide, were purchased from the suppliers. 1,3,5-Tris(4-nitrophenyl)benzene (TNPB)[1], 2,4,6-tris(4-nitrophenyl)-1,3,5-triazine (TNPT)[2] and 2,4,6-tris(4-aminophenyl)-1,3,5-triazine (TAPT)[2] were synthesized by the procedure described in the literature.

1,3,5-Tris(4-nitrophenyl)benzene (TNPB): Yield 77 %.

IR (ATR) $\tilde{\upsilon}$ / cm^{-1}: 1663, 1592, 1508, 1341, 1105, 841, 748, 689.

^{13}C CP/MAS NMR (400 MHz) δ / ppm: 145.7, 138.6, 129.9, 127.8, 125.8, 121.8.

2,4,6-Tris(4-nitrophenyl)-1,3,5-triazine (TNPT): Yield 85 %.

IR (ATR) $\tilde{\upsilon}$ / cm^{-1}: 1531, 1414, 1334, 1105, 1010, 824, 743, 684.

^{13}C CP/MAS NMR (400 MHz) δ / ppm: 169.3, 149.8, 140.0, 131.0, 123.2.

2,4,6-Tris(4-aminophenyl)-1,3,5-triazine (TAPT): Yield 76 %.

IR (ATR) $\tilde{\upsilon}$ / cm^{-1}: 3460, 3319, 3208, 1604, 1493, 1428, 1362, 1177, 1147, 1128, 811, 722, 676.

^{1}H NMR (400 MHz, DMSO-d$_6$) δ / ppm: 8.35 (d, 6H, J = 8.6 Hz), 6.68 (d, 6H, J = 8.6 Hz), 5.90 (s, 6H).

^{13}C NMR (100 MHz, DMSO-d$_6$) δ / ppm: 170.0, 153.4, 130.6, 123.3, 113.5.

[1] Li, G.; Wang, Z. Microporous Polyimides with Uniform Pores for Adsorption and Separation of CO_2 Gas and Organic Vapors. *Macromolecules* **2013**, *46*, 3058−3066.

[2] Halder, A.; Kandambeth, S.; Biswal, B.P.; Kaur, G.; Roy, N.C.; Addicoat, M.; Salunke, J.K.; Banerjee, S.; Vanka, K.; Heine, T.; Verma, S.; Banerjee, R. Decoding the Morphological Diversity in Two Dimensional Crystalline Porous Polymers by Core Planarity Modulation. *Angew. Chem. Int. Ed.* **2016**, *55*, 7806–7810.

1.1. Synthesis of AZO-B-P1 − AZO-T-P14

Synthesis of AZO-B-P1

Yield 50 %.

IR (ATR) $\tilde{\upsilon}$ / cm^{-1}: 3356, 1593, 1509, 1448, 1392, 1346, 841.

^{13}C CP/MAS NMR (400 MHz) δ / ppm: 151.9, 142.7, 127.7, 123.3.

Elemental Analysis: 69.93 %C (calc. 83.46), 10.14 %N (calc. 12.17).

Synthesis of AZO-T-P2

Yield 37 %.

IR (ATR) $\tilde{\upsilon}$ / cm^{-1}: 3384, 1584, 1509, 1437, 1410, 1359, 819.

^{13}C CP/MAS NMR (400 MHz) δ / ppm: 170.0, 154.3, 137.8, 122.7.

Elemental Analysis: 70.55 %C (calc. 72.40), 22.95 %N (calc. 24.12).

Synthesis of AZO-T-P3

Yield 52 %.

IR (ATR) $\tilde{\upsilon}$ / cm^{-1}: 3353, 1583, 1504, 1435, 1409, 1359, 816.

^{13}C CP/MAS NMR (400 MHz) δ / ppm: 170.0, 154.0, 137.9, 122.7, 114.1.

Elemental Analysis: 60.72 %C (calc. 72.40), 18.84 %N (calc. 24.12).

Synthesis of AZO-B-P4

Yield 78 %.

IR (ATR) $\tilde{\upsilon}$ / cm^{-1}: 3356, 1591, 1509, 1446, 1390, 1342, 1106, 841, 748, 690.

^{13}C CP/MAS NMR (400 MHz) δ / ppm: 151.7, 146.6, 128.0, 123.1, 115.3.

Elemental Analysis: 57.84 %C (calc. 80.16), 9.59 %N (calc. 15.58).

Synthesis of AZO-T-P5

AZO-T-P5 was synthesized by the similar procedure described in the literature.[3] TNPT (500 mg, 1.13 mmol), 1,4-phenylenediamine (184 mg, 1.70 mmol), DMF (50 mL) and KOH (634 mg, 11.3 mmol) were added in double-necked flask and heated to reflux under N_2 atmosphere. After 24 h, the reaction mixture was cooled to room temperature, poured in 300 mL of distilled water and stirred for 1 h. The reaction mixture was filtered off and washed with hot distilled water, acetone and THF. After drying at 140 °C under vacuum for 5 h, black solid was obtained (360 mg, yield 70 %).

IR (ATR) $\tilde{\upsilon}$ / cm^{-1}: 3376, 1583, 1492, 1406, 1356, 1336, 811.

^{13}C CP/MAS NMR (600 MHz) δ / ppm: 169.0, 153.0, 149.5, 137.6, 128.6, 122.5, 115.1.

Elemental Analysis: 67.64 %C (calc. 71.67), 20.04 %N (calc. 24.76).

Synthesis of AZO-B-P6

AZO-B-P6 was synthesized by the similar procedure described in the literature.[3] TNPB (500 mg, 1.13 mmol), benzidine (313 mg, 1.70 mmol), DMF (50 mL) and KOH (634 mg, 11.3 mmol) were added in double-necked flask and heated to reflux under N_2 atmosphere. After 24 h, the reaction mixture was cooled to room temperature, poured in 300 mL of distilled water and stirred for 1 h. The reaction mixture was filtered off and washed with hot distilled water, acetone and THF. After drying at 140 °C under vacuum for 5 h, black solid was obtained (242 mg, yield 41 %).

IR (ATR) $\tilde{\upsilon}$ / cm^{-1}: 3361, 3030, 1590, 1513, 1441, 1388, 1342, 833.

^{13}C CP/MAS NMR (400 MHz) δ / ppm: 151.5, 146.3, 141.6, 127.6, 115.5.

Elemental Analysis: 71.26 %C (calc. 82.26), 10.35 %N (calc. 13.32).

Synthesis of AZO-B-P7

AZO-B-P7 was synthesized by the similar procedure described in the literature.[3] TNPB (500 mg, 1.13 mmol), 4,4'-diaminodiphenylmethane (340 mg, 1.70 mmol), DMF (50 mL) and KOH (634 mg, 11.3 mmol) were added in double-necked flask and heated to reflux under N_2 atmosphere. After 24 h, the reaction mixture was cooled to room temperature, poured in 300 mL of distilled water and stirred for 1 h. The reaction mixture was filtered off and washed with hot distilled water, acetone and THF. After drying at 140 °C under vacuum for 5 h, black solid was obtained (175 mg, yield 29 %).

IR (ATR) $\tilde{\upsilon}$ / cm^{-1}: 3358, 1590, 1510, 1443, 1388, 1341, 832.

---

[3] Patel, H.A.; Je, S.H.; Park, J.; Jung, Y.; Coskun, A.; Yavuz, C.T. Directing the Structural Features of N_2-Phobic Nanoporous Covalent Organic Polymers for CO_2 Capture and Separation. *Chem. Eur. J.* **2014**, *20*, 772–780.

^{13}C CP/MAS NMR (400 MHz) δ / ppm: 151.5, 146.4, 141.6, 128.5, 123.8, 115.4, 40.1.

Elemental Analysis: 71.88 %C (calc. 82.35), 10.46 %N (calc. 12.98).

Synthesis of AZO-B-P8

AZO-B-P8 was synthesized by the similar procedure described in the literature.[3] TNPB (500 mg, 1.13 mmol), 4,4'-oxydianiline (343 mg, 1.70 mmol), DMF (50 mL) and KOH (634 mg, 11.3 mmol) were added in double-necked flask and heated to reflux under N_2 atmosphere. After 24 h, the reaction mixture was cooled to room temperature, poured in 300 mL of distilled water and stirred for 1 h. The reaction mixture was filtered off and washed with hot distilled water, acetone and THF. After drying at 140 °C under vacuum for 5 h, black solid was obtained (430 mg, yield 70 %).

IR (ATR) $\tilde{\upsilon}$ / cm^{-1}: 3353, 1590, 1494, 1444, 1390, 1341, 827.

^{13}C CP/MAS NMR (400 MHz) δ / ppm: 151.9, 146.5, 142.8, 127.6, 123.3, 115.8.

Elemental Analysis: 70.55 %C (calc. 79.83), 11.12 %N (calc. 12.93).

Synthesis of AZO-B-P9

AZO-B-P9 was synthesized by the similar procedure described in the literature.[3] TNPB (500 mg, 1.13 mmol), 4,4'-ethylenedianiline (361 mg, 1.70 mmol), DMF (50 mL) and KOH (634 mg, 11.3 mmol) were added in double-necked flask and heated to reflux under N_2 atmosphere. After 24 h, the reaction mixture was cooled to room temperature, poured in 300 mL of distilled water and stirred for 1 h. The reaction mixture was filtered off and washed with hot distilled water, acetone and THF. After drying at 140 °C under vacuum for 5 h, black solid was obtained (384 mg, 61 %).

IR (ATR) $\tilde{\upsilon}$ / cm^{-1}: 3359, 2923, 2851, 1590, 1512, 1441, 1390, 1342, 825.

^{13}C CP/MAS NMR (400 MHz) δ / ppm: 151.7, 146.5, 141.2, 128.4, 123.8, 115.3, 37.0.

Elemental Analysis: 72.44% C (calc. 82.44), 10.64 %N (calc. 12.65).

Synthesis of AZO-B-P10

AZO-B-P10 was synthesized by the similar procedure described in the literature.[3] TNPB (500 mg, 1.13 mmol), 4,4'-diaminobenzophenone (364 mg, 1.70 mmol), DMF (50 mL) and KOH (634 mg, 11.3 mmol) were added in double-necked flask and heated to reflux under N_2 atmosphere. After 24 h, the reaction mixture was cooled to room temperature, poured in 300 mL of distilled water and stirred for 1 h. The reaction mixture was filtered off and washed with hot distilled water, acetone and THF. After drying at 140 °C under vacuum for 5 h, black solid was obtained (241 mg, 39 %).

IR (ATR) $\tilde{\upsilon}$ / cm^{-1}: 3342, 1587, 1512, 1434, 1391, 1346, 829.

^{13}C CP/MAS NMR (400 MHz) δ / ppm: 194.7, 151.9, 141.5, 129.8, 114.2.

Elemental Analysis: 74.64 %C (calc. 80.27), 11.23 %N (calc. 12.65).

Synthesis of AZO-B-P11

AZO-B-P11 was synthesized by the similar procedure described in the literature.[3] TNPB (500 mg, 1.13 mmol), 4,4'-diaminodiphenyl sulfide (367 mg, 1.70 mmol), DMF (50 mL) and KOH (634 mg, 11.3 mmol) were added in double-necked flask and heated to reflux under N_2 atmosphere. After 24 h, the reaction mixture was cooled to room temperature, poured in 300 mL of distilled water and stirred for 1 h. The reaction mixture was filtered off and washed with hot distilled water, acetone and THF. After drying at 140 °C under vacuum for 5 h, black solid was obtained (197 mg, yield 31 %).

IR (ATR) $\tilde{\upsilon}$ / cm^{-1}: 3364, 1590, 1510, 1444, 1391, 1341, 840.

^{13}C CP/MAS NMR (400 MHz) δ / ppm: 151.5, 145.9, 139.6, 127.5, 124.1, 116.3.

Elemental Analysis: 71.77 %C (calc. 77.54), 10.58 %N (calc. 12.56).

Synthesis of AZO-T-P12

AZO-T-P12 was synthesized by the similar procedure described in the literature.[3] TNPT (500 mg, 1.13 mmol), benzidine (310 mg, 1.70 mmol), DMF (50 mL) and KOH (634 mg, 11.3 mmol) were added in double-necked flask and heated to reflux under N_2 atmosphere. After 24 h, the reaction mixture was cooled to room temperature, poured in 300 mL of distilled water and stirred for 1 h. The reaction mixture was filtered off and washed with hot distilled water, acetone and THF. After drying at 140 °C under vacuum for 5 h, black solid was obtained (410 mg, yield 69 %).

IR (ATR) $\tilde{\upsilon}$ / cm^{-1}: 3368, 1582, 1503, 1494, 1435, 1407, 1358, 812.

^{13}C CP/MAS NMR (400 MHz) δ / ppm: 169.6, 154.0, 137.1, 128.7, 122.5, 114.9.

Elemental Analysis: 70.36 %C (calc. 74.99), 20.63 %N (calc. 21.20).

Synthesis of AZO-T-P13

AZO-T-P13 was synthesized by the similar procedure described in the literature.[3] TNPT (500 mg, 1.13 mmol), 4,4'-diaminodiphenylmethane (330 mg, 1.70 mmol), DMF (50 mL) and KOH (634 mg, 11.3 mmol) were added in double-necked flask and heated to reflux under N_2 atmosphere. After 24 h, the reaction mixture was cooled to room temperature, poured in 300 mL of distilled water and stirred for 1 h. The reaction mixture was filtered off

and washed with hot distilled water, acetone and THF. After drying at 140 °C under vacuum for 5 h, black solid was obtained (321 mg, yield 52 %).

IR (ATR) $\tilde{\upsilon}$ / cm^{-1}: 3344, 1582, 1495, 1435, 1407, 1355, 811.

^{13}C CP/MAS NMR (400 MHz) δ / ppm: 170.1, 154.0, 145.6, 138.2, 129.6, 122.6, 115.5, 40.2.

Elemental Analysis: 67.72 %C (calc. 75.26), 16.73 %N (calc. 20.65).

Synthesis of AZO-T-P14

AZO-T-P14 was synthesized by the similar procedure described in the literature.[3] TNPT (500 mg, 1.13 mmol), 4,4'-oxydianiline (340 mg, 1.70 mmol), DMF (50 mL) and KOH (634 mg, 11.3 mmol) were added in double-necked flask and heated to reflux under N_2 atmosphere. After 24 h, the reaction mixture was cooled to room temperature, poured in 300 mL of distilled water and stirred for 1 h. The reaction mixture was filtered off and washed with hot distilled water, acetone and THF. After drying at 140 °C under vacuum for 5 h, black solid was obtained (334 mg, yield 54 %).

IR (ATR) $\tilde{\upsilon}$ / cm^{-1}: 3384, 1601, 1493, 1409, 1336, 824.

^{13}C CP/MAS NMR (400 MHz) δ / ppm: 169.5, 150.4, 139.7, 130.2, 123.4, 116.9.

Elemental Analysis: 71.59 %C (calc. 72.78), 18.03 %N (calc. 20.58).

2. FT-IR spectra

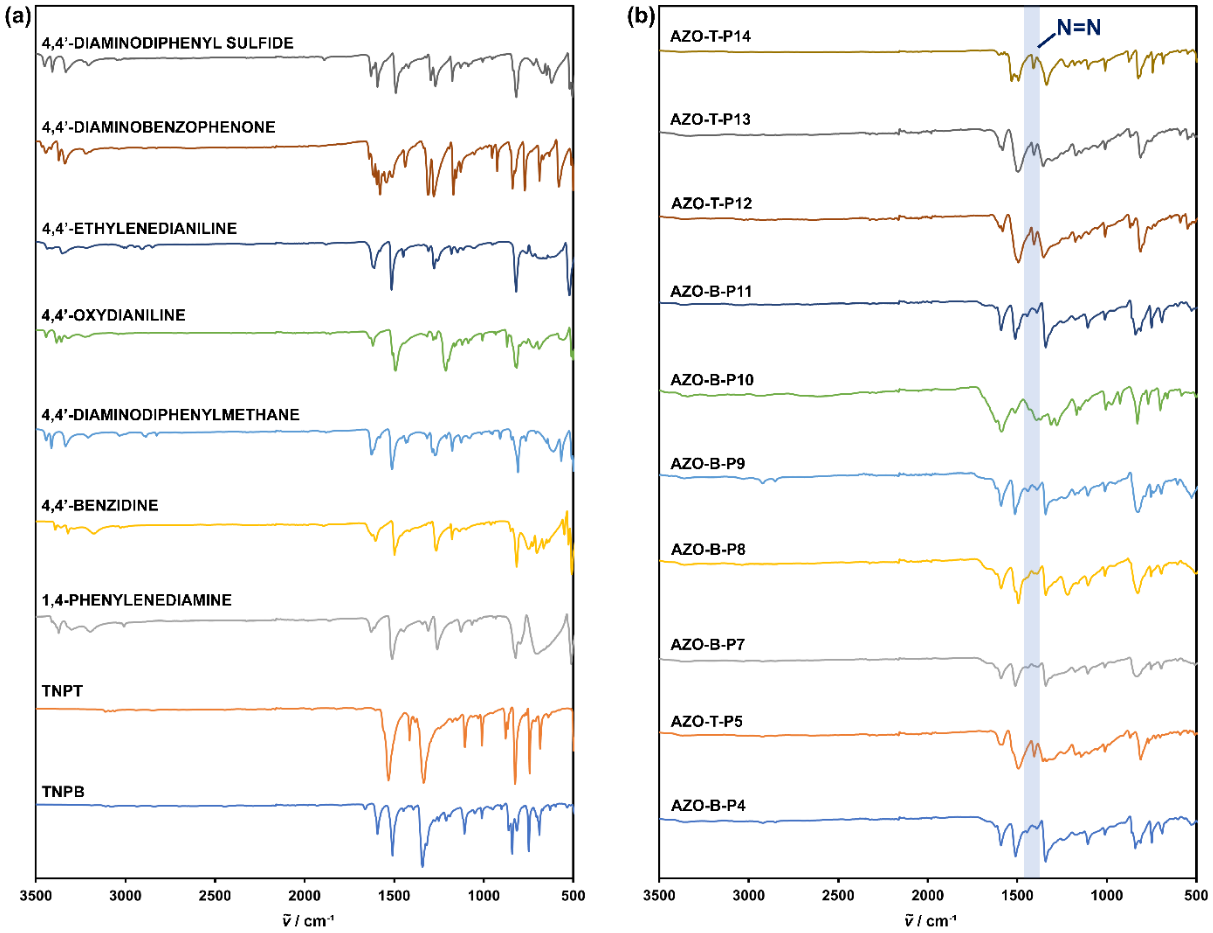

Figure S1. FT-IR spectra of (a) starting aromatic nitro and amino monomers and (b) azo-bridged polymers.

3. ^{13}C CP/MAS NMR spectra

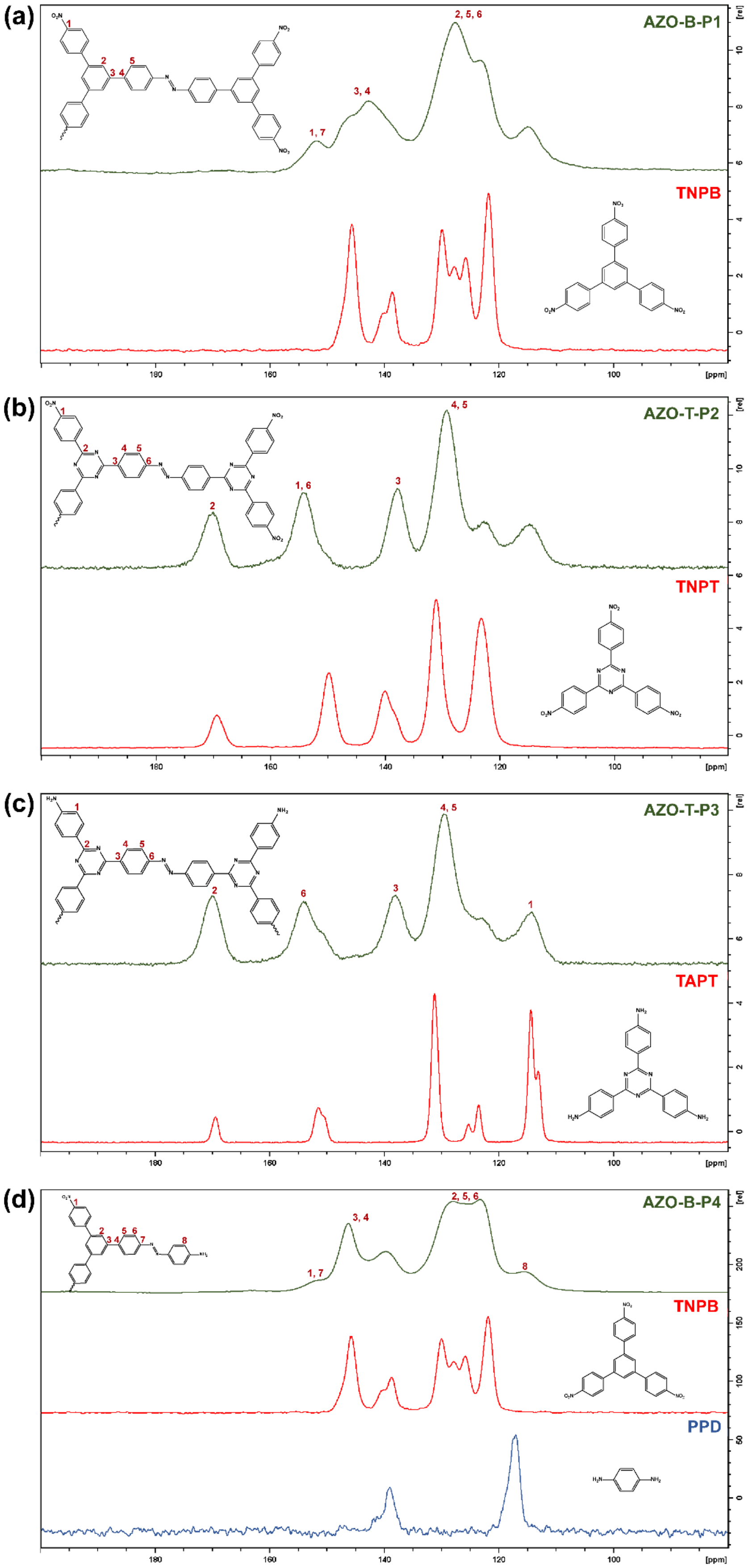

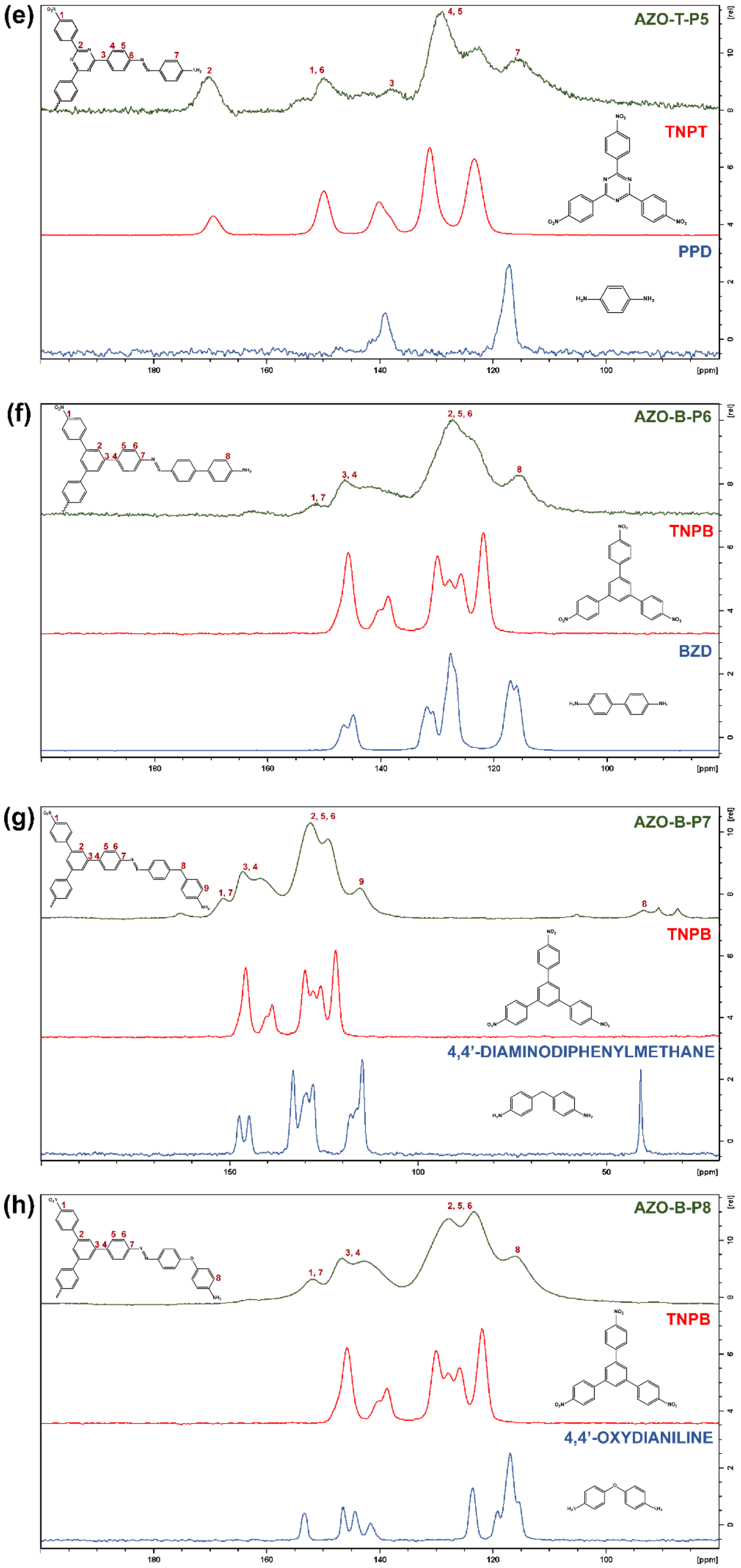
(e)
AZO-T-P5
TNPT
PPD
(f)
AZO-B-P6
TNPB
BZD
(g)
AZO-B-P7
TNPB
4,4'-DIAMINODIPHENYLMETHANE
(h)
AZO-B-P8
TNPB
4,4'-OXYDIANILINE
[ppm]

(i)

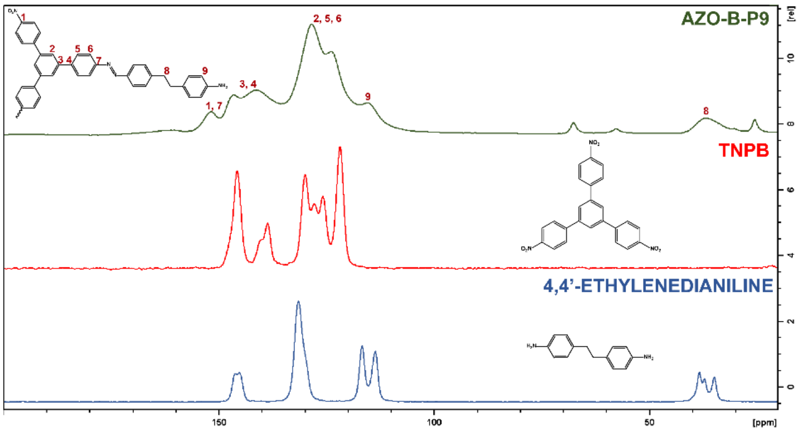
AZO-B-P9
TNPB
4,4'-ETHYLENEDIANILINE
2, 5, 6
3, 4
1, 7
9
8
150
100
50
[ppm]
[rel]

(j)

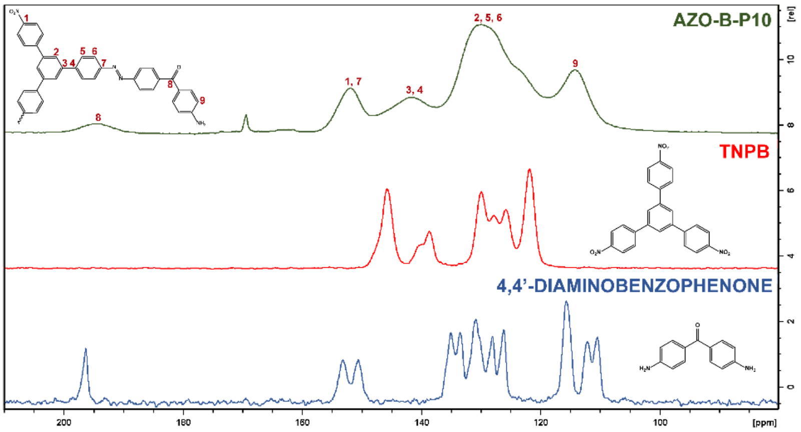
AZO-B-P10
TNPB
4,4'-DIAMINOBENZOPHENONE
2, 5, 6
1, 7
3, 4
9
8
200
180
160
140
120
100
[ppm]
[rel]

(k)

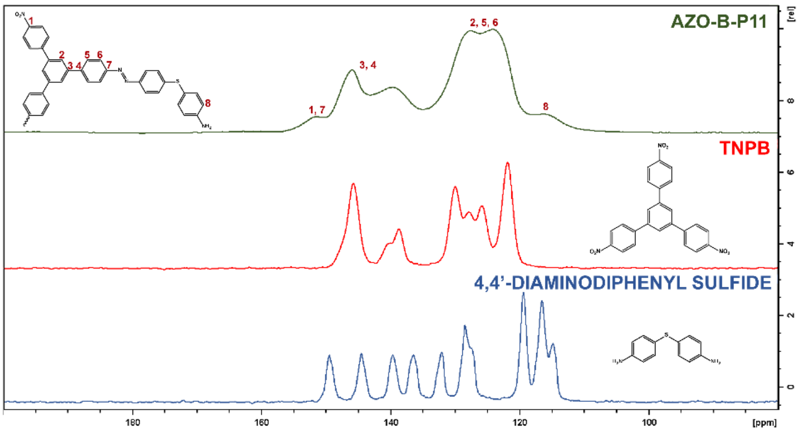
AZO-B-P11
TNPB
4,4'-DIAMINODIPHENYL SULFIDE
2, 5, 6
3, 4
1, 7
8
180
160
140
120
100
[ppm]
[rel]

(l)

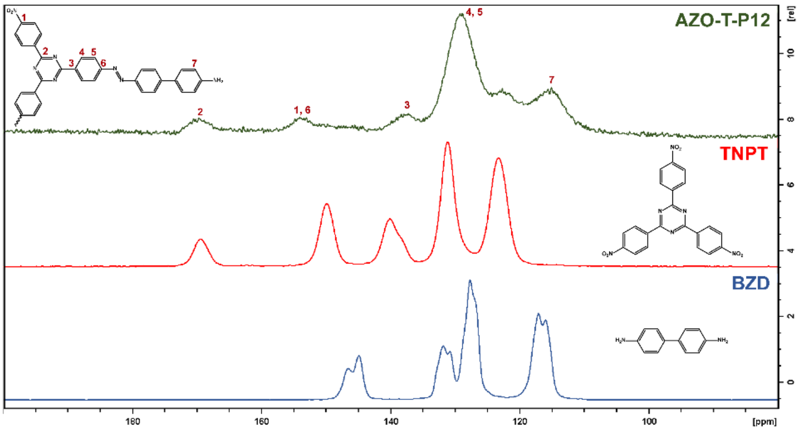
AZO-T-P12
TNPT
BZD
4, 5
7
2
1, 6
3
180
160
140
120
100
[ppm]
[rel]

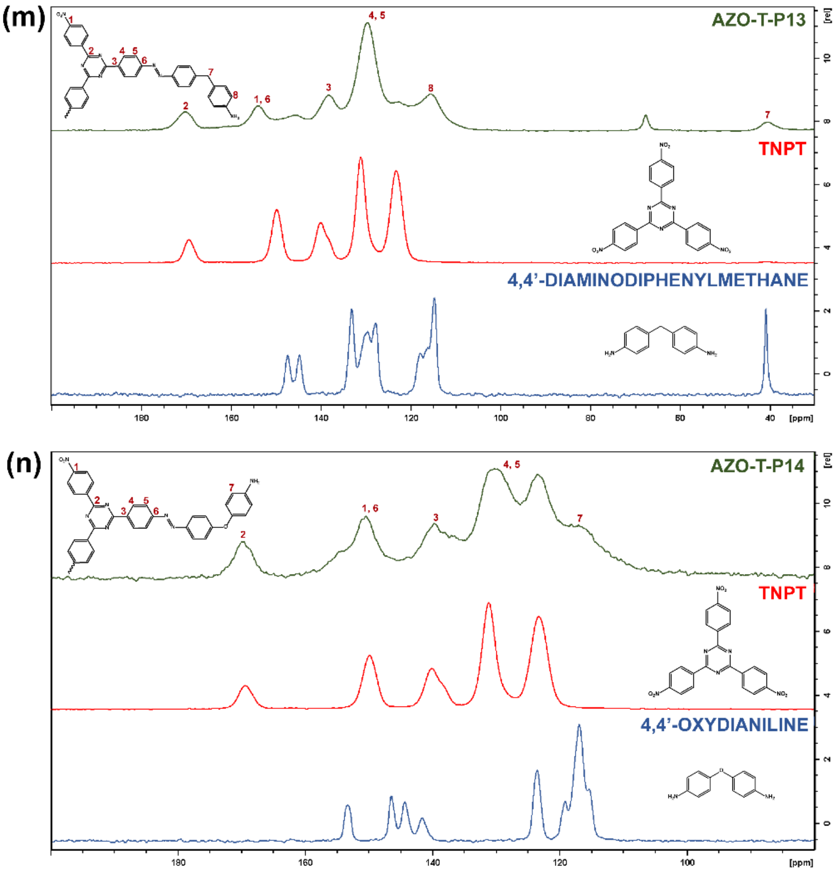

Figure S2. Comparison of ^{13}C CP/MAS NMR spectra of azo-bridged polymers and corresponding starting aromatic nitro and amino monomers.

4. Powder X-ray diffraction

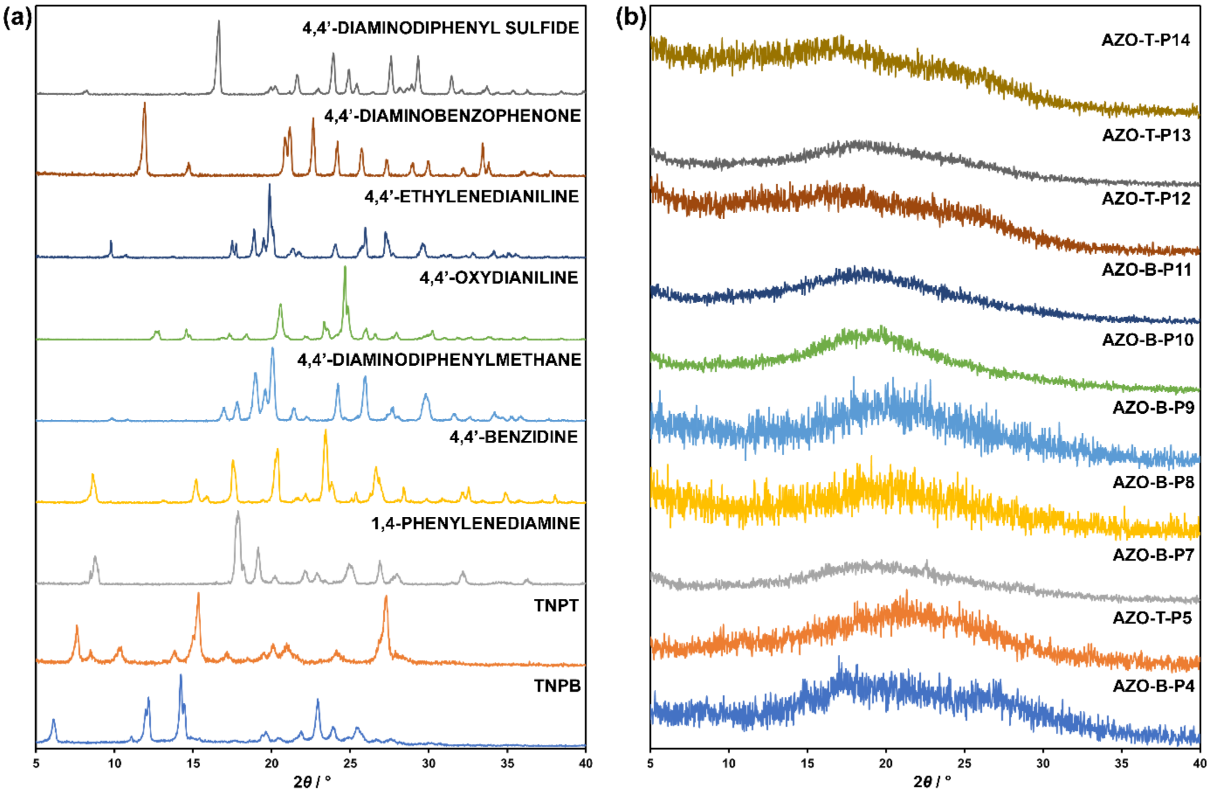

Figure S3. PXRD diffractograms of (a) starting aromatic nitro and amino monomers and (b) azo-bridged polymers.

5. Thermogravimetric analysis

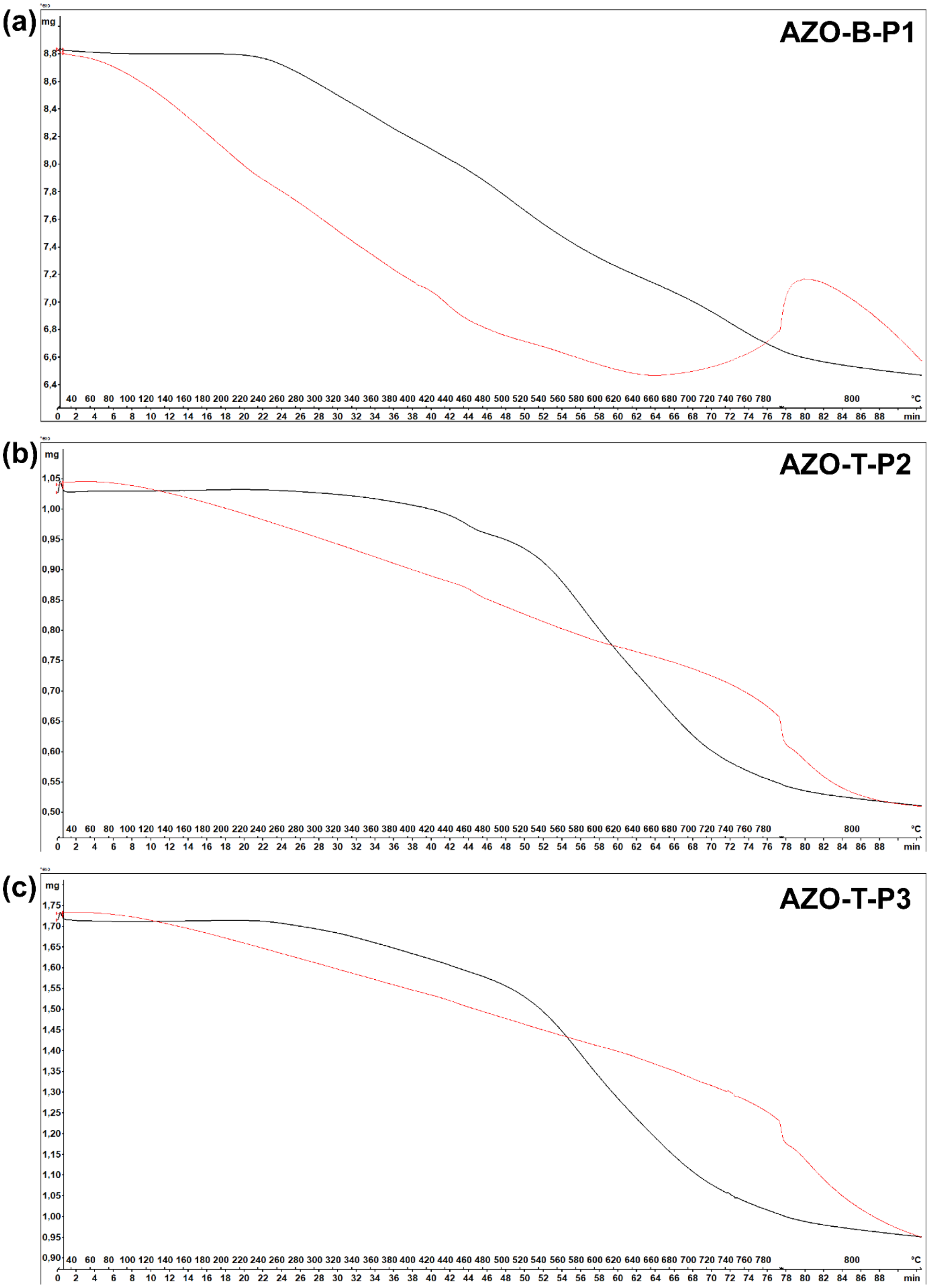
(a)
AZO-B-P1
(b)
AZO-T-P2
(c)
AZO-T-P3
mg
°C
min

(d)

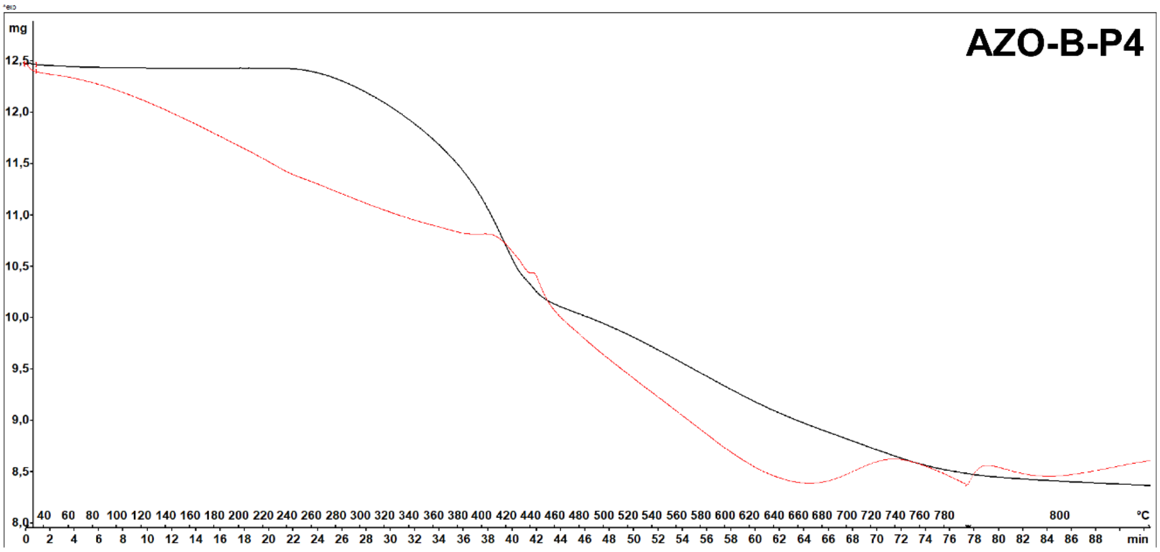

(e)

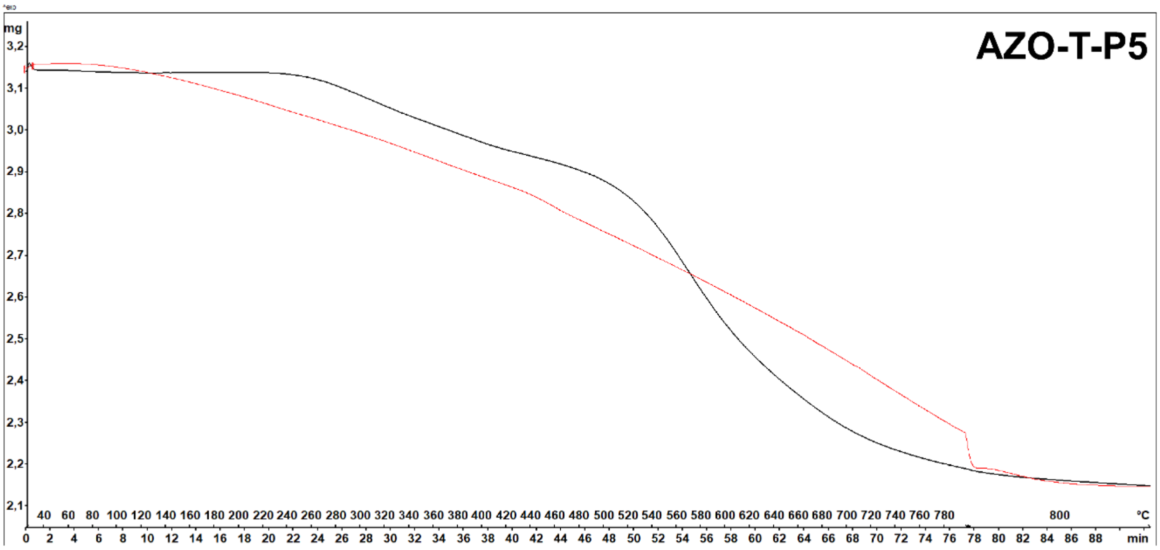

(f)

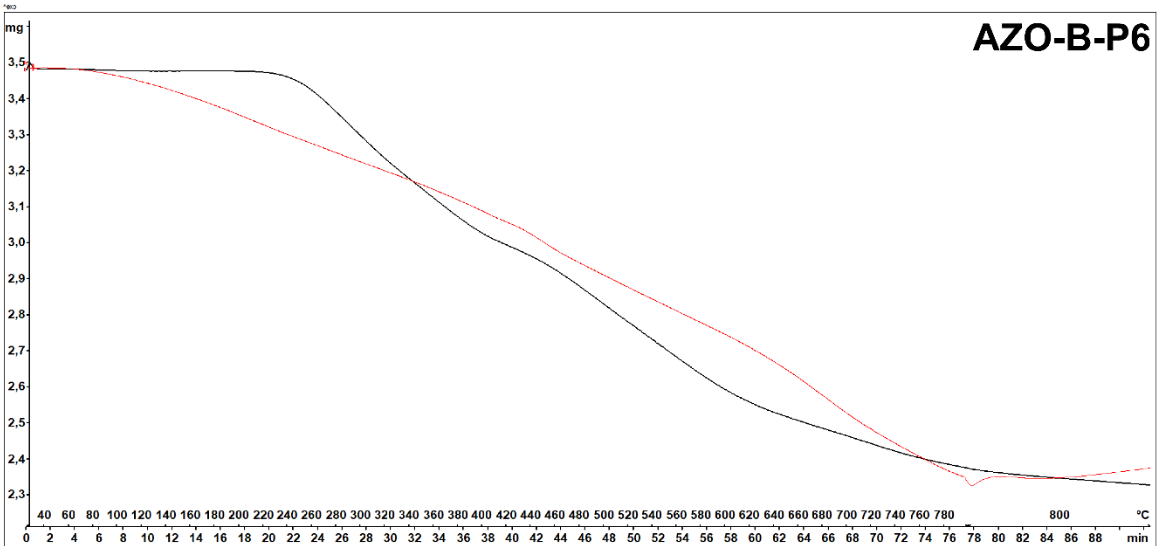

(g)

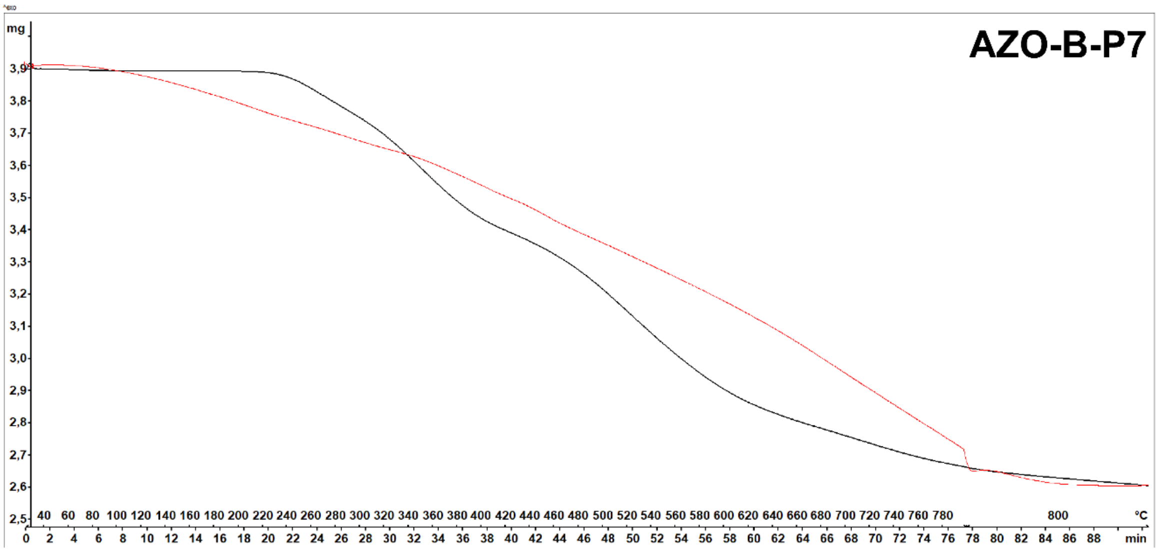
AZO-B-P7
mg
3,9
3,8
3,7
3,6
3,5
3,4
3,3
3,2
3,1
3,0
2,9
2,8
2,7
2,6
2,5
40 60 80 100 120 140 160 180 200 220 240 260 280 300 320 340 360 380 400 420 440 460 480 500 520 540 560 580 600 620 640 660 680 700 720 740 760 780 800 °C
0 2 4 6 8 10 12 14 16 18 20 22 24 26 28 30 32 34 36 38 40 42 44 46 48 50 52 54 56 58 60 62 64 66 68 70 72 74 76 78 80 82 84 86 88 min

(h)

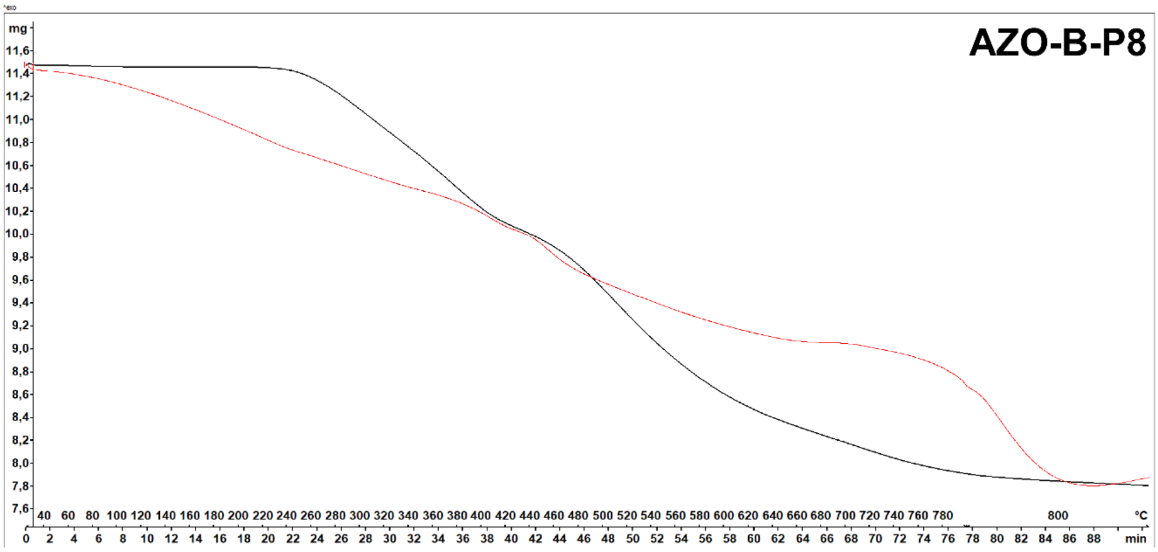
AZO-B-P8
mg
11,6
11,4
11,2
11,0
10,8
10,6
10,4
10,2
10,0
9,8
9,6
9,4
9,2
9,0
8,8
8,6
8,4
8,2
8,0
7,8
7,6
40 60 80 100 120 140 160 180 200 220 240 260 280 300 320 340 360 380 400 420 440 460 480 500 520 540 560 580 600 620 640 660 680 700 720 740 760 780 800 °C
0 2 4 6 8 10 12 14 16 18 20 22 24 26 28 30 32 34 36 38 40 42 44 46 48 50 52 54 56 58 60 62 64 66 68 70 72 74 76 78 80 82 84 86 88 min

(i)

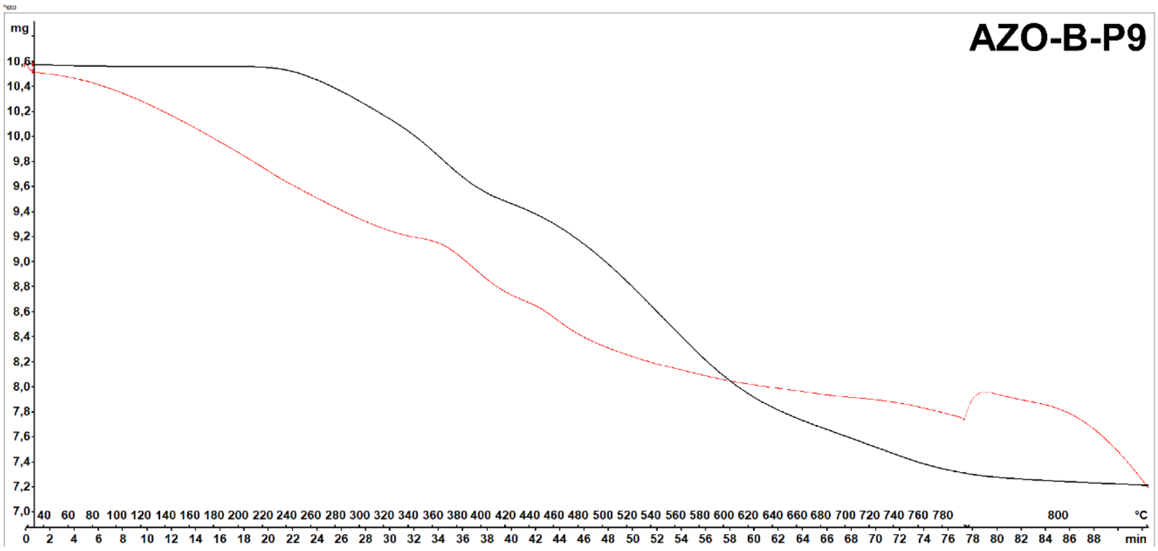
AZO-B-P9
mg
10,6
10,4
10,2
10,0
9,8
9,6
9,4
9,2
9,0
8,8
8,6
8,4
8,2
8,0
7,8
7,6
7,4
7,2
7,0
40 60 80 100 120 140 160 180 200 220 240 260 280 300 320 340 360 380 400 420 440 460 480 500 520 540 560 580 600 620 640 660 680 700 720 740 760 780 800 °C
0 2 4 6 8 10 12 14 16 18 20 22 24 26 28 30 32 34 36 38 40 42 44 46 48 50 52 54 56 58 60 62 64 66 68 70 72 74 76 78 80 82 84 86 88 min

(j)

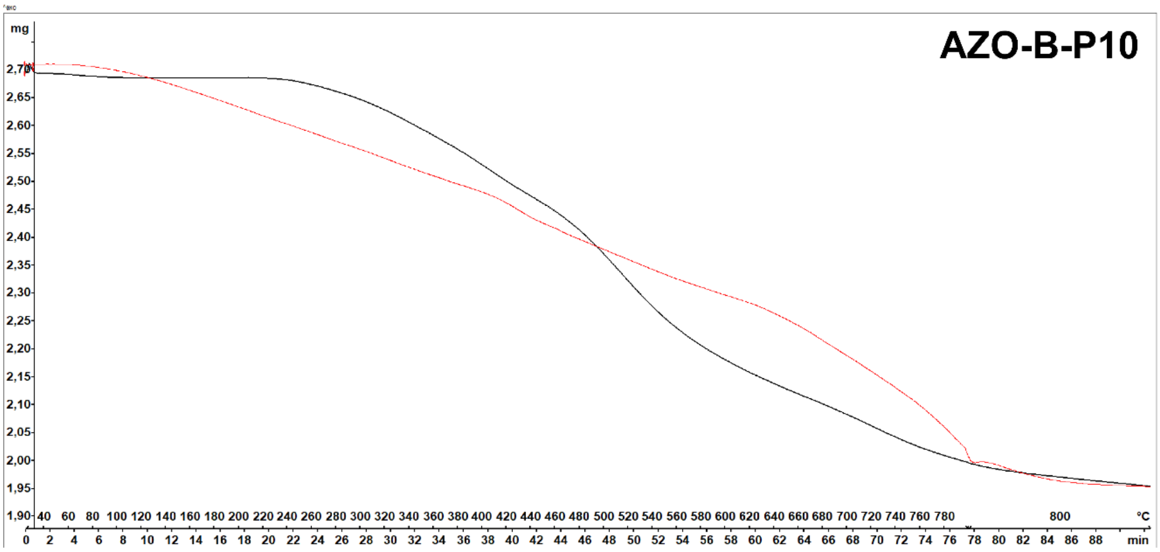
AZO-B-P10
mg
2,70 2,65 2,60 2,55 2,50 2,45 2,40 2,35 2,30 2,25 2,20 2,15 2,10 2,05 2,00 1,95 1,90
40 60 80 100 120 140 160 180 200 220 240 260 280 300 320 340 360 380 400 420 440 460 480 500 520 540 560 580 600 620 640 660 680 700 720 740 760 780 800 °C
0 2 4 6 8 10 12 14 16 18 20 22 24 26 28 30 32 34 36 38 40 42 44 46 48 50 52 54 56 58 60 62 64 66 68 70 72 74 76 78 80 82 84 86 88 min

(k)

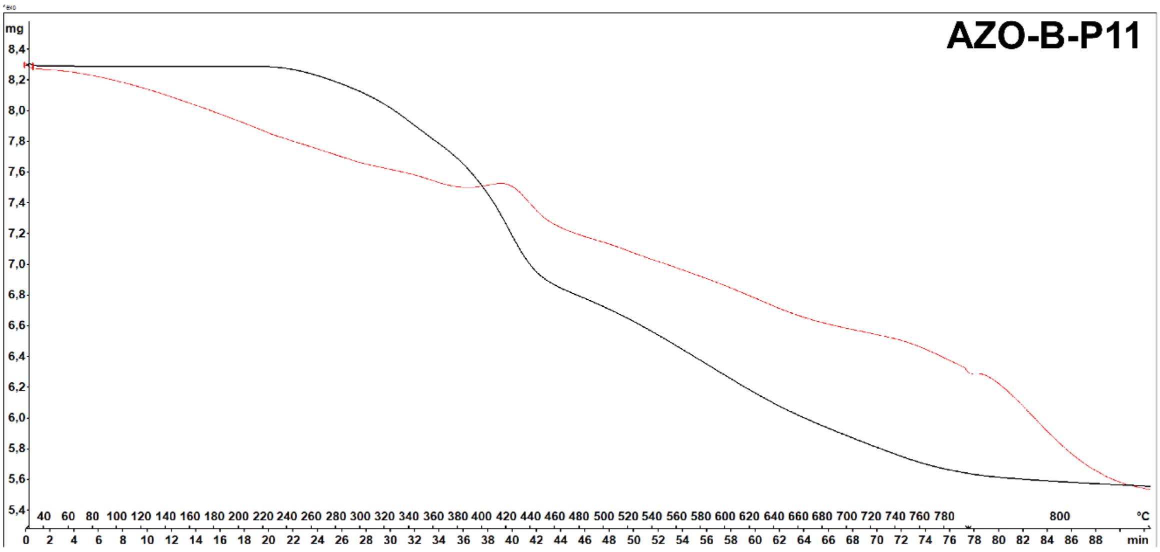
AZO-B-P11
mg
8,4 8,2 8,0 7,8 7,6 7,4 7,2 7,0 6,8 6,6 6,4 6,2 6,0 5,8 5,6 5,4
40 60 80 100 120 140 160 180 200 220 240 260 280 300 320 340 360 380 400 420 440 460 480 500 520 540 560 580 600 620 640 660 680 700 720 740 760 780 800 °C
0 2 4 6 8 10 12 14 16 18 20 22 24 26 28 30 32 34 36 38 40 42 44 46 48 50 52 54 56 58 60 62 64 66 68 70 72 74 76 78 80 82 84 86 88 min

(l)

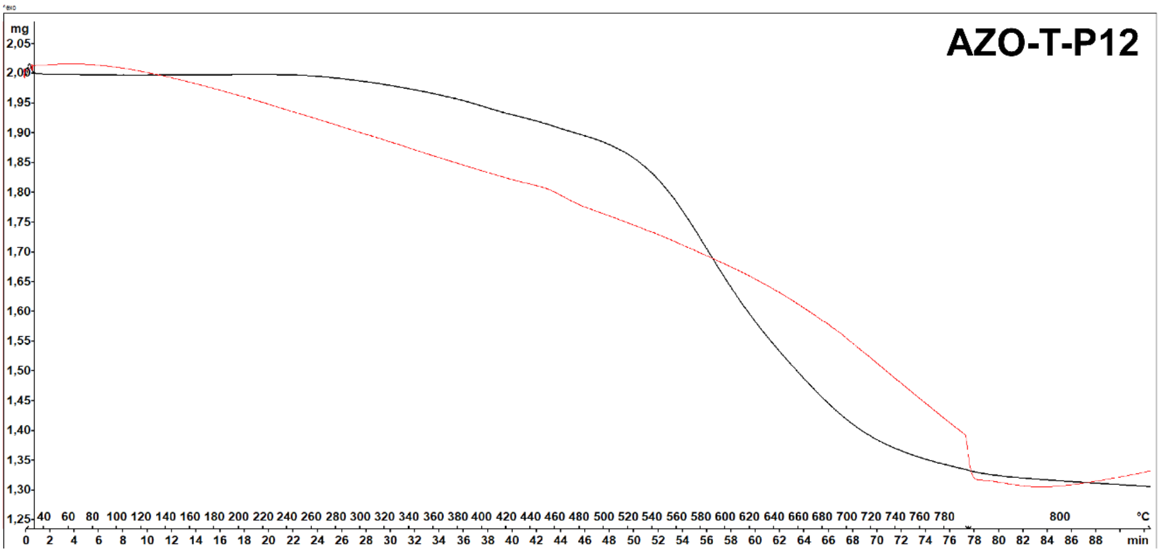
AZO-T-P12
mg
2,05 2,00 1,95 1,90 1,85 1,80 1,75 1,70 1,65 1,60 1,55 1,50 1,45 1,40 1,35 1,30 1,25
40 60 80 100 120 140 160 180 200 220 240 260 280 300 320 340 360 380 400 420 440 460 480 500 520 540 560 580 600 620 640 660 680 700 720 740 760 780 800 °C
0 2 4 6 8 10 12 14 16 18 20 22 24 26 28 30 32 34 36 38 40 42 44 46 48 50 52 54 56 58 60 62 64 66 68 70 72 74 76 78 80 82 84 86 88 min

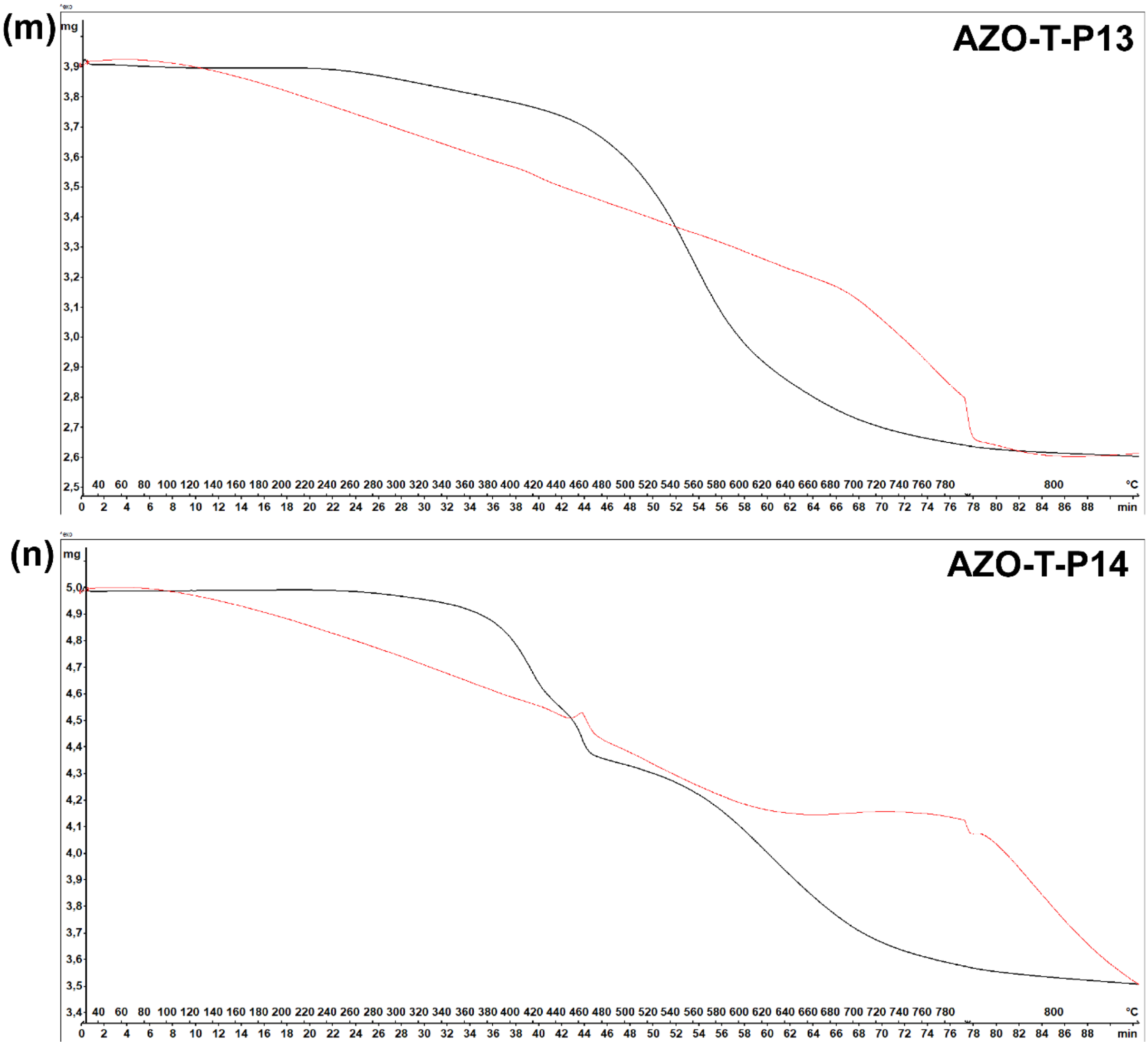

Figure S4. TGA (black) and DSC (red) curves of azo-bridged polymers.

6. Computational studies of azo-bridged polymers

Table S1. Unit cell parameters (a, b, c, α, β and γ) of the optimized geometries (PBE-D3/pob-TZVP-rev2) of benzene-based (AZO-B) and triazine-based (AZO-T) model compounds.

	**Compound**	**Space group**	**CRYSTAL17 label**	***a*** **/ Å**	***b*** **/ Å**	***c*** **/ Å**	α **/ °**	β **/ °**	γ **/ °**
**AA stacking**	AZO-B	$P\bar{3}$	147	25.6247	25.6247	3.5830	90	90	120
	AZO-B-PPD	$P\bar{3}$	147	36.1836	36.1836	3.5759	90	90	120
	AZO-B-BZD	$P3$	143	43.3587	43.3587	3.5786	90	90	120
	AZO-T	$P\bar{3}$	147	25.2081	25.2081	3.5170	90	90	120
	AZO-T-PPD	$P\bar{3}$	147	35.6570	35.6570	3.5265	90	90	120
	AZO-T-BZD	$P3$	143	42.7213	42.7213	3.5440	90	90	120

Table S2. Calculated framework properties of the AA stacked 2D layered compounds (framework density, available pore volume and average surface area) and the size of the simulation box used in the GCMC calculations.

	**Compound**	**GCMC simulation box**	**Framework density, g/cm^3**	**Available pore volume, cm^3/g**	**Average surface area, m^2/g**
**AA stacking**	AZO-B	2×2×8	0.563	1.140	1957
	AZO-B-PPD	2×2×8	0.411	1.794	2237
	AZO-B-BZD	2×2×8	0.351	2.203	2306
	AZO-T	2×2×8	0.598	1.031	1828
	AZO-T-PPD	2×2×8	0.431	1.671	2127
	AZO-T-BZD	2×2×8	0.367	2.075	2207

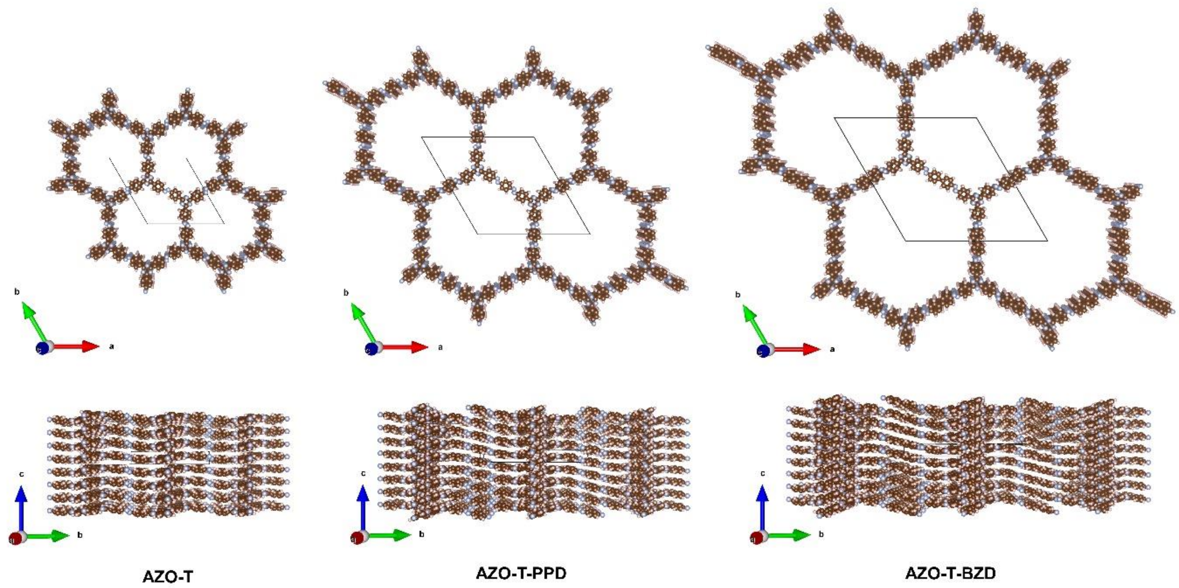

Figure S5. Optimized geometries (PBE-D3/pob2-TZVP-rev2) of AZO-T, AZO-T-PPD and AZO-T-BZD with eclipsed (AA-stacked) geometries of 2D layers shown along the *c* and *a* unit cell vectors). Unit cells are represented by grey lines.

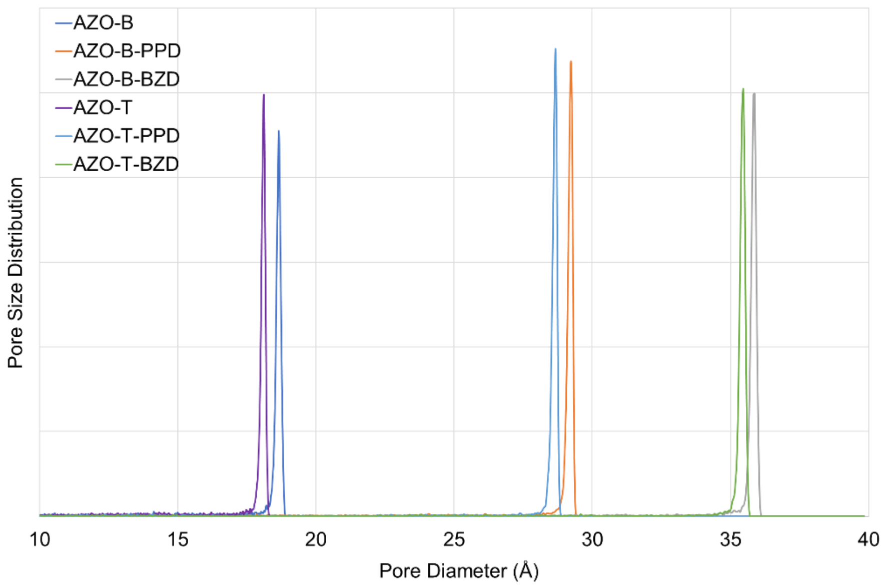

Figure S6. Pores size distribution of AA stacked configurations.

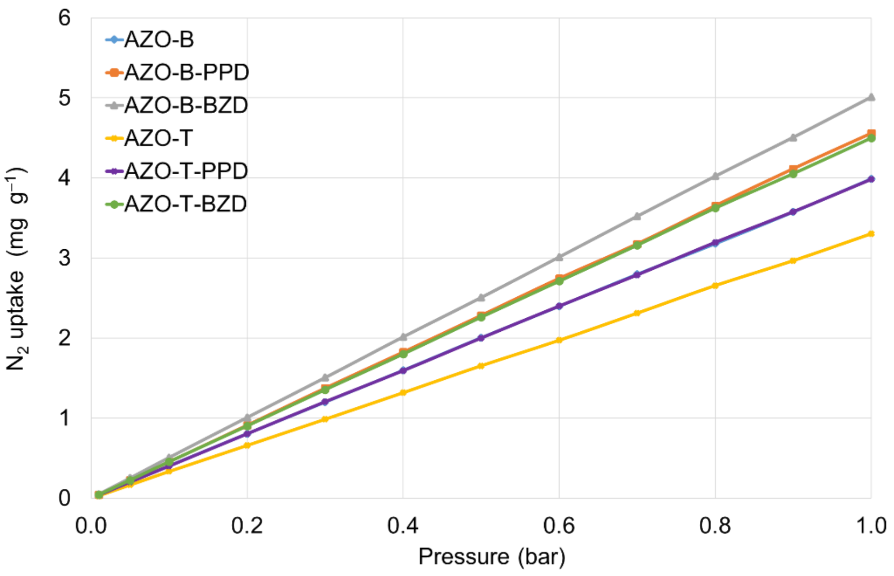

Figure S7. N_2 adsorption isotherms simulated at 298 K.
